# Transcriptomic and Neuroimaging Decoding of Brain‐Immune Crosstalk in Thyroid Eye Disease

**DOI:** 10.1002/advs.202523609

**Published:** 2026-03-05

**Authors:** Haiyang Zhang, Yuting Liu, Shufan Jiang, Zilin Fang, Mengda Jiang, Xiaofeng Tao, Tianyi Zhu, Jipeng Li, Sijie Fang, Xuefei Song, Yinwei Li, Jing Sun, Chuanjun Tong, Zhengrun Gao, Huifang Zhou, Xianqun Fan

**Affiliations:** ^1^ State Key Laboratory of Eye Health Department of Ophthalmology Shanghai Ninth People's Hospital Shanghai Jiao Tong University School of Medicine Shanghai China; ^2^ Shanghai Key Laboratory of Orbital Diseases and Ocular Oncology Shanghai China; ^3^ Department of Radiology Shanghai Ninth People's Hospital Shanghai Jiao Tong University School of Medicine Shanghai China; ^4^ Institute of Neuroscience Center For Excellence in Brain Science and Intelligence Technology Chinese Academy of Sciences Shanghai China; ^5^ Songjiang Research Institute Songjiang Hospital affiliated to Shanghai Jiao Tong University School of Medicine Shanghai China

**Keywords:** allen human brain atlas, autoimmune thyroid disease, brain‐immune crosstalk, functional magnetic resonance imaging, thyroid eye disease, transcriptomics

## Abstract

Autoimmune thyroid diseases (AITD) are systemic conditions frequently associated with neurological manifestations, yet the underlying neural and immunological mechanisms remain unclear. This study focuses on thyroid eye disease (TED), a representative AITD, to provide a deeper insight into its neural mechanism. We first combined resting‐state functional magnetic resonance imaging (rs‐fMRI) data from a retrospective cohort of 116 TED patients with transcriptomic data from the Allen Human Brain Atlas. The analysis of rs‐fMRI data demonstrated significant alterations in frontal, parietal, subcortical, and brainstem regions in TED. By integrating rs‐fMRI data with regional transcriptomic profiles derived from the postmortem Allen Human Brain Atlas, enabling region‐level transcriptional inference, we revealed enriched pathways related to synaptic signaling, neurovascular regulation, and immune activation. Tissue and cellular level enrichment further showed close association with the cortex and neurons. Key neuroimaging findings identified in the retrospective cohort were subsequently validated in an independent prospective cohort of TED patients and healthy controls (TED: 39; HC: 42) using paired rs‐fMRI and peripheral blood RNA sequencing data, which identified significant associations between immune cell infiltration and neural activity patterns. Collectively, these findings delineate coordinated brain–immune associations in TED and generate hypotheses regarding neuroimmune interactions in AITD.

## Graphical abstract

This study employed an imaging transcriptomics framework integrating resting‐state fMRI with Allen Human Brain Atlas transcriptomic data, coupled with peripheral blood RNA sequencing, to decode brain‐immune crosstalk in thyroid eye disease. Frontal, parietal, subcortical, and brainstem regions were identified as key neuroimmune‐ vulnerable regions, where neuroimaging changes aligned with molecular processes enriched in synaptic signaling, neurovascular regulation, and immune activation. Independent cohort validation confirmed coordinated neural‐immune associations in this autoimmune thyroid disease.

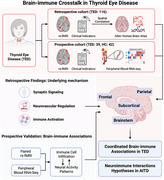

## 1 Introduction

Autoimmune thyroid diseases (AITD), including Graves’ disease, Hashimoto's thyroiditis, and thyroid eye disease (TED), are common organ‐specific autoimmune diseases, with an estimated prevalence rate of 5% in the general population [1]. Moreover, the prevalence of subclinical individuals with autoantibodies may be even higher [2]. In addition to endocrine disorders, AITD patients suffer neuropsychiatric symptoms such as anxiety, depression, and cognition dysfunction, which cannot be entirely attributed to thyroid hormone abnormalities [3]. Emerging evidence suggests that peripheral immune disorders may exert an impact on the central nervous system [4]. Mechanistically, the autoimmune response mediated by circulating autoantibodies and infiltrating immune cells may not only impair thyroid function, but also affect brain function through systemic inflammation in AITD [5, 6]. Thus, it is of great importance to explore the brain‐immune crosstalk mechanism in AITD to elucidate the systemic features and uncover potential therapeutic targets.

Transcriptomics and neuroimaging serve as critical tools to explore underlying molecular alteration and brain involvement, respectively [7, 8]. Recent studies have revealed the transcription abnormalities in genes related to thyroid function and immune modulation, highlighting the systemic inflammatory response in AITD [9, 10]. Meanwhile, neuroimaging has been employed to unveil brain alterations in AITD, in which disease‐related brain regions were found implicated in cognition and emotion [11]. Notably, brain alterations displayed associations with peripheral immune biomarkers, indicating potential crosstalk between systemic inflammation and central dysfunction in AITD [12]. However, the cellular or molecular basis underlying brain‐immune crosstalk still remains elusive due to the lack of specificity of neuroimaging, and the absence of cross‐modal joint analysis of high‐dimensional data [13]. Thus, integrating neuroimaging and transcriptomics is necessary to link macroscopic neuroimaging findings with underlying cellular and molecular alterations at the transcriptomic scale in AITD.

As a typical subtype of AITD, TED is characterized by a pronounced systemic inflammatory response, and shared autoantibody‐mediated mechanisms with other AITD entities, including Graves’ disease and Hashimoto's thyroiditis [14]. TED patients generally exhibit neuropsychiatric disorders in addition to orbital symptoms, which suggests the brain involvement of an AITD nature [15]. Importantly, the inflammatory and immune processes observed in TED reflect systemic autoimmunity rather than being confined to orbital tissues, making it a suitable model for interrogating brain‐immune interactions at the systemic level. Given that the orbit and the brain are intimately interconnected in immune response due to the shared vascular and lymphatic circuit, TED is an ideal model for studying brain‐immune crosstalk in AITD [16]. While certain orbital manifestations are TED‐specific, the neuroimmune associations investigated in this study are expected to capture immune‐mediated mechanisms common across AITD.

**FIGURE 1:**
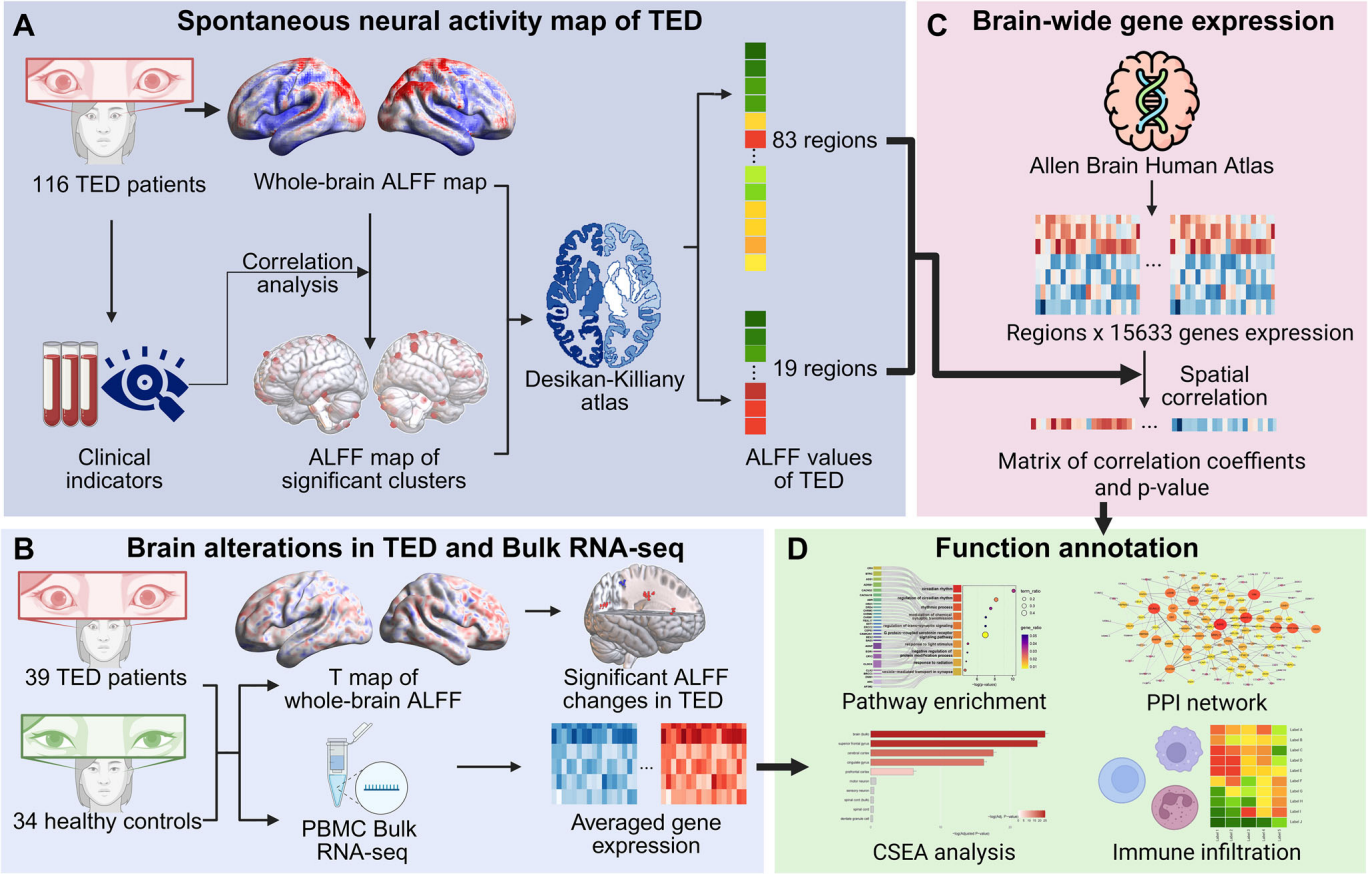
Overview of the study pipeline. (A) Spontaneous neural activity mapping in TED. Whole‐brain ALFF maps were obtained from the TED brain test dataset. Correlation analysis was performed between ALFF values and clinical indicators. ALFF values were extracted using two strategies based on the Desikan–Killiany atlas: (1) whole‐brain parcellation resulting in an 83‐region ALFF matrix, and (2) a targeted approach focusing on 19 ROIs identified from correlation analyses. (B) Brain‐wide gene expression analysis. The Allen Human Brain Atlas was utilized to extract gene expression data for the identified brain regions. Spatial correlation analysis generated a matrix of correlation coefficients and p‐values. (C) Brain alterations in TED and bulk RNA sequencing. Comparison of whole‐brain ALFF between the TED brain‐blood dataset and the HC brain‐blood dataset identified significant ALFF changes. Bulk RNA‐seq from PBMC samples was used to analyze transcriptomic differences. (D) Functional annotation. Pathway enrichment, protein–protein interaction network analysis, CSEA analysis, and immune infiltration assessments were conducted to explore the biological significance of the identified genes. This figure was created in BioRender. **Abbreviations**: TED, thyroid eye disease; ALFF, amplitude of low‐frequency fluctuations; DK, Desikan–Killiany atlas; ROI, region of interest; AHBA, Allen Human Brain Atlas; PBMC, peripheral blood mononuclear cell; RNA‐seq, RNA sequencing; PPI, protein–protein interaction; CSEA, cell‐type specific enrichment analysis.

Therefore, we integrated resting state functional MRI (rs‐fMRI) and blood peripheral transcriptomics to reveal the molecular basis underlying brain alterations in TED. Using the *TED brain test dataset* containing rs‐fMRI data, Allen Human Brain Atlas (AHBA) was utilized to associate disease‐related brain regions with corresponding transcriptomic signatures in this retrospective patient cohort. To validate the observed changes, we prospectively collected and analyzed rs‐fMRI and peripheral blood transcriptomic data from a group of TED patients and healthy controls (HC), forming the *TED brain‐blood dataset* and *HC brain‐blood dataset*. These datasets comprised paired neuroimaging and peripheral blood RNA‐sequencing data. From a technical perspective, this multimodal framework aims to bridge macroscale functional brain alterations with immune‐related molecular signatures. From a biological perspective, it provides potential support to the underlying mechanism of neuroimmune involvement in AITD‐associated brain dysfunction. Study pipeline is demonstrated in Figure 1. The detailed participant recruitment and screening process is shown in Figure S1.

## 2 Results

### 2.1 Frontal, Parietal, Subcortical, and Brainstem Regions as Potential Disease‐Related Brain Regions in TED

**FIGURE 2:**
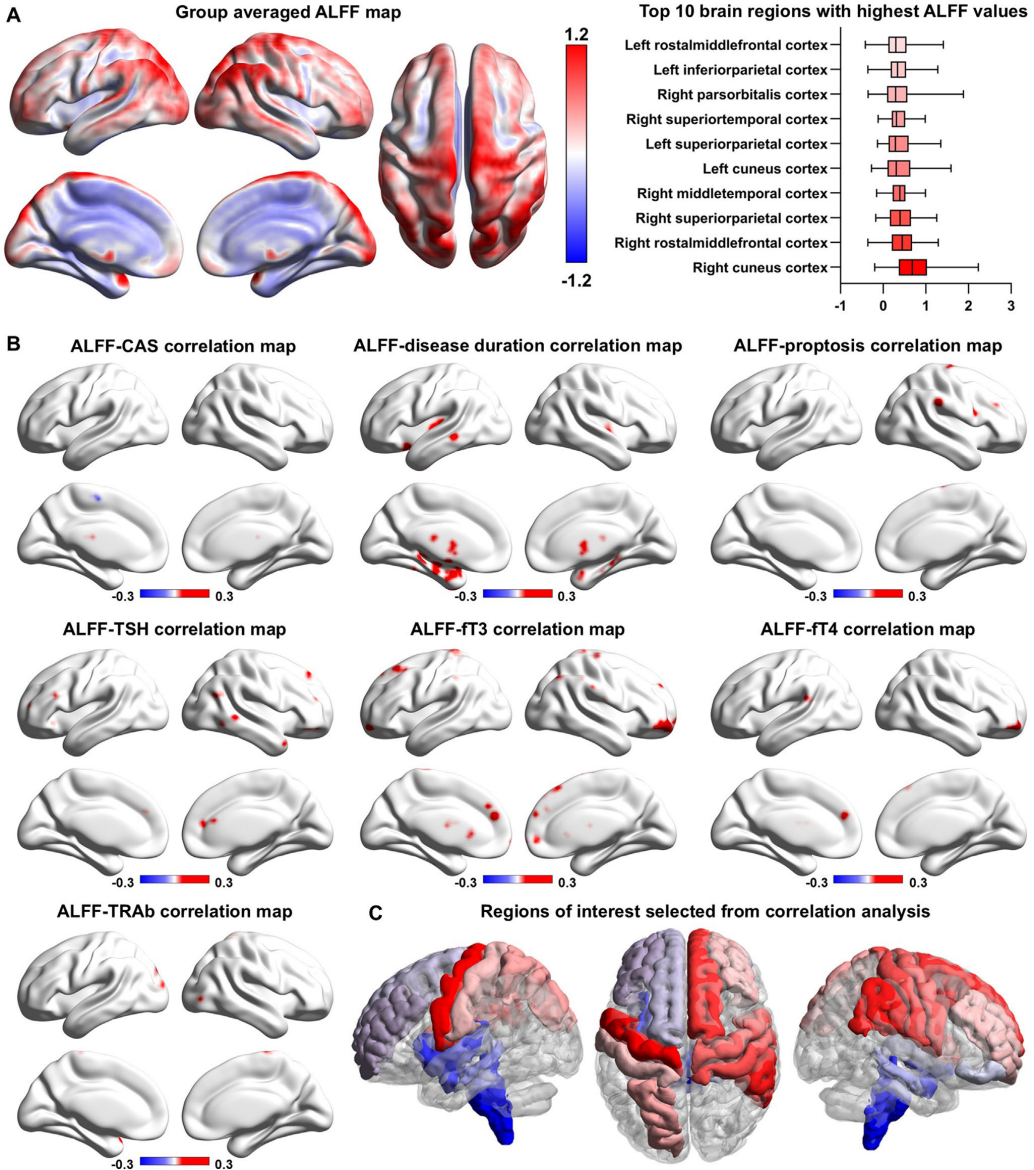
Regional neural activity patterns and clinical correlations in TED. (A) Group‐averaged ALFF map of TED brain test dataset. Warmer colors (red) indicate higher ALFF values, while cooler colors (blue) represent lower ALFF values. The right panel lists the top 10 brain regions with the highest ALFF values in TED patients. (B) The R‐map depicting correlations between various clinical parameters and ALFF values in the TED brain test dataset (voxel‐level *p*<0.005, cluster‐level *p*<0.05, GRF corrected). (C) Regions of interest mapped onto the Desikan–Killiany atlas. Significant clusters from (A) were overlaid on the Desikan–Killiany atlas, and any parcellation with ≥6 voxels in a given cluster were defined as a region of interestR. This panel shows the DK atlas regions corresponding to these regions of interest. **Abbreviations**: ALFF, amplitude of low‐frequency fluctuations; CAS, clinical activity score; fT3, free triiodothyronine; fT4, free thyroxine; TED, thyroid eye disease; TRAb, thyrotropin recrptor antibody; TSH thyroid‐stimulating hormone; GRF, Gaussian random field.

First, using rs‐fMRI data from the TED brain test dataset, we examined patterns of resting‐state spontaneous neural activity via amplitude of low‐frequency fluctuation (ALFF) analysis. The demographic and clinical profiles of the patients are shown in Table 1. Group‐averaged ALFF maps revealed prominent activity in multiple cortical regions, with the frontal, parietal, and temporal lobes showing the highest ALFF values (Figure 2A). Specifically, the top 10 regions with the highest ALFF values included the cuneus (occipital), rostral middle frontal (frontal), and superior parietal cortex (parietal), highlighting these areas as predominant sites of TED‐related neural activity.

**TABLE 1:** Demographic and clinical profiles of included patients.

	*TED brain test dataset* (n = 116)	*TED brain‐blood dataset* (n = 39)	*HC brain‐blood dataset* (n = 42)
Age (years)	45.39±12.73	46.13±14.63	43.76±14.96
Sex (male/female)	42/74	17/22	20/24
Disease duration (months)	12.00 (7.000, 29.25)	15.00 (7.000, 33.00)	—
CAS	2.000 (1.000, 3.000)	1.000 (0.000, 2.500)	—
Proptosis (mm)	19.52±2.640	19.75±2.710	—
Comordity of AITD			
Grave's disease	104	36	—
Hashimoto's thyroiditis	11	2	—
Others	1	0	
TSH (µIU/mL)	1.480 (0.3500, 2.810)	1.061 (0.1665, 2.490)	—
fT3 (pg/mL)	3.705 (3.110, 4.410)	4.115 (3.483, 4.818)	—
fT4 (ng/dL)	6.600±7.405	7.965±7.009	—
TRAb (IU/L)	8.228±9.609	7.812±9.958	—
Continuous variables are presented as the mean (± standard deviation) for normally distributed data, or as the median (interquartile range) for non‐normally distributed data. Categorical variables are presented as counts. Group comparison was conducted using a two‐sample t‐test. TED: thyroid eye disease; HC: healthy controls; CAS: clinical activity score; AITD: autoimmune thyroid diseases; TSH: thyroid‐stimulating hormone; fT3: free triiodothyronine; fT4: free thyroxine; TRAb: thyroid‐stimulating hormone receptor antibodies			

To explore the clinical relevance of ALFF distribution in TED, we performed voxel‐wise correlation analyses between ALFF values and multiple clinical indicators (Figure 2B, Supplementary Results, Figure S2, Table S1). The spatial distribution of clinical associations was primarily localized to the frontal lobe, subcortical areas, and brainstem. For non‐blood‐based clinical indicators related to TED, significant associations were found primarily in subcortical regions, including the bilateral hippocampus and the left thalamus, as well as in the brainstem, the frontal lobe—particularly the right rostral middle frontal cortex—and the temporal lobe, with prominent involvement of the left inferior temporal cortex. Bilateral insular cortices also exhibited significant associations, while the parietal lobe showed limited involvement. For serum thyroid function and auto‐antibody parameters, the most significant brain regions associated with ALFF were concentrated in the frontal lobe (particularly the right rostral middle frontal cortex, right superior frontal cortex, left superior frontal cortex, and left rostral middle frontal cortex), the parietal and temporal lobes, with minor involvement of subcortical regions, occipital lobe, and brainstem.

To identify spatially meaningful regions, clusters with significant ALFF‐clinical correlations were overlaid on the Desikan–Killiany (DK) atlas (Figure 2C). Brain regions containing ≥6 voxels within a significant cluster were selected as regions of interest (ROIs), resulting in a set of 19 TED‐related brain regions (Table S2). These ROIs were primarily located in the frontal lobes (e.g., rostral middle frontal, superior frontal), parietal regions (e.g., postcentral, supramarginal), and subcortical structures (e.g., hippocampus, thalamus), as well as the brainstem. The identified ROIs may represent key neural substrates underlying TED pathogenesis and were selected for subsequent imaging‐transcriptomic analyses. The preliminary discoveries may also indicate the neural activity pattern in AITD.

### 2.2 Brain Transcriptional Signatures Related to Spontaneous Neural Activity Across Brain Regions

**FIGURE 3:**
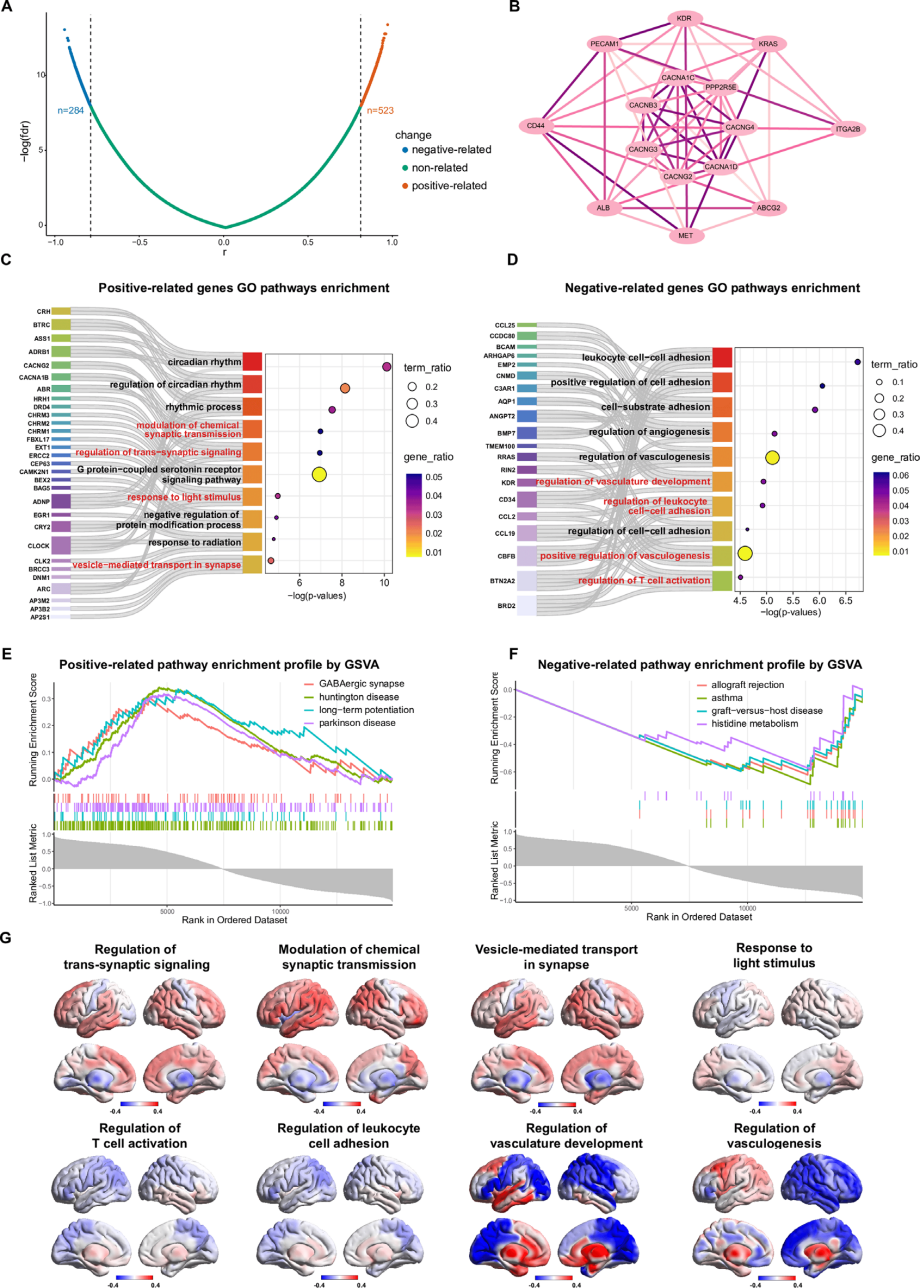
Gene analysis correlated with ALFF values across brain regions. (A) Volcano plot depicting genes significantly correlated with ALFF values. Genes with significant correlations (FDR‐corrected p < 0.05, |r| > 0.8) are highlighted. (B) PPI network for genes positively correlated with ALFF values, constructed using the STRING database and analyzed with the MCODE algorithm in Cytoscape. Nodes represent proteins, and edges denote interactions between them. The edge intensity reflects the STRING combined score, indicating the confidence level of each interaction. (C) Pathway enrichment analysis of positively correlated genes using GO terms (adjusted p < 0.05). The graph illustrates the top 10 enriched GO terms. (D) Pathway enrichment analysis of negatively correlated genes using GO terms (adjusted p < 0.05). The graph illustrates the top 10 enriched GO terms. (E) GSVA of genes correlated with ALFF values, highlighting the top four positively enriched pathways. (F) GSVA of genes correlated with ALFF values, highlighting the top four negatively enriched pathways. (G) Voxel‐wise correlation maps displaying associations between whole‐brain ALFF and selected pathways, as determined by the ssGSEA algorithm. **Abbreviations**: ALFF, amplitude of low‐frequency fluctuations; FDR, false discovery rate; MCODE, molecule complex detection; PPI, protein–protein interaction; GO, Gene Ontology; GSVA, gene set variation analysis; ssGSEA, single‐sample gene set enrichment analysis.

To elucidate genetic changes associated with spontaneous neural activity in TED patients, we identified genes significantly correlated with ALFF (voxel‐level *p* < 0.005, cluster‐level *p* < 0.05, Gaussian random field [GRF] corrected) across 15,632 genes from the AHBA, applying a false discovery rate (FDR)‐corrected threshold (*p* < 0.05, |r| > 0.8). This analysis yielded 523 positively correlated genes and 284 negatively correlated genes (Figure 3A).

Protein–protein interaction (PPI) network analysis revealed a core module of 14 genes linking ALFF‐associated neural hyperactivity in TED patients, primarily enriched in calcium channel subunits (e.g., CACNA1C, CACNB3) and vascular‐related molecules (e.g., PECAM1, CD44) (Figure 3B; Figures S3 and S4). Gene Ontology (GO) enrichment analysis indicated that positively correlated genes were predominantly associated with synaptic signaling and light stimulus response, including “modulation of chemical synaptic transmission”, “regulation of trans‐synaptic signaling”, “response to light stimulus”, and “vesicle‐mediated transport in synapse” (Figure 3C; Figure S5). Kyoto Encyclopedia of Genes and Genomes (KEGG) enrichment analysis further validated the positively correlated genes were related with “dopaminergic synapse”, “calcium signaling pathway”, “neurotrophin signaling pathway”, and “serotonergic synapse” (Figure S6). In contrast, negatively correlated genes were enriched in vascular development and immune regulation, such as “regulation of vasculature development”, “leukocyte cell–cell adhesion”, “positive regulation of vasculogenesis”, and “T cell activation” (Figure 3D; Figure S7). KEGG revealed similar pathway enrichment in “cell adhesion molecules”, “primary immunodeficiency”, and “allograft rejection” (Figure S8). Gene Set Variation Analysis (GSVA) revealed similar patterns, with positively correlated genes linked to synaptic signaling and neurodegenerative diseases, while negatively correlated genes were associated with immune response and histidine metabolism (Figure 3E,F).

In addition, the single sample Gene Set Enrichment Analysis (ssGSEA) revealed distinct regional enrichment patterns of these pathways across cortical and subcortical areas (Figure 3G). Positively correlated genes were enriched in synaptic vesicle transport within cortical regions, such as the left superior frontal gyrus (0.345) and right inferior parietal cortex (0.362), whereas subcortical regions, like pallidum (−0.495) and caudate (−0.430), showed the opposite pattern. Immune‐related pathways showed limited cortical involvement but pronounced subcortical heterogeneity, with T cell activation positively associated with the right insula (0.190), while leukocyte adhesion was negatively correlated with the left postcentral gyrus (−0.151). Vasculogenesis‐related pathways displayed pronounced regional heterogeneity, being highly enriched in the left amygdala (0.793) and entorhinal cortex (0.791), but negatively correlated with the right cuneus (−0.896) and superior temporal gyrus (−0.763). Additionally, subcortical nuclei (e.g., thalamus, putamen) exhibited opposing roles, being positively linked to vascular regulation but negatively associated with synaptic processes (Table S3). These findings highlight a functional gradient in TED patients, with cortical regions are predominantly involved in synaptic and immune modulation, whereas subcortical regions exhibit stronger associations with vascular processes.

### 2.3 Transcriptional Profiles and Spatial Enrichment of Neuronal Subtypes Associated with ALFF Correlated Genes

**FIGURE 4:**
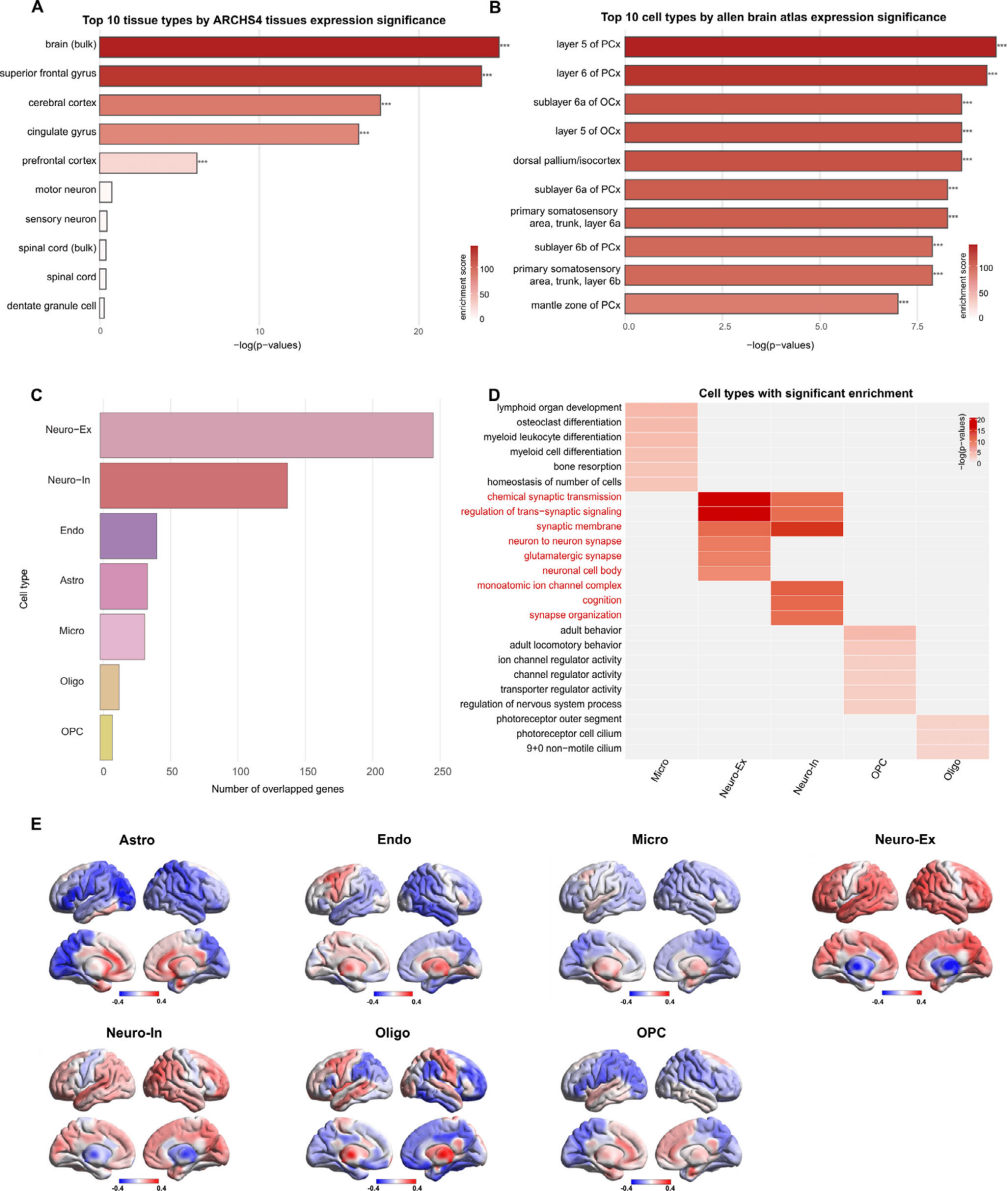
Transcriptional signatures of specific tissue and cell types for positively regulated genes. (A) Significant tissue‐type enrichment was identified using the ARCHS4 dataset. (B) Significant cell‐type enrichment was identified from the AHBA. (C) Number of overlapping genes with positively related genes across seven canonical cell types. (D) Heatmap of enriched pathways across cell types using GO terms (adjusted *p* < 0.05) (E) Voxel‐wise correlation maps displaying associations between whole‐brain ALFF and seven canonical cell types, as determined by the ssGSEA algorithm. *Significant correlations are marked with asterisks: *p ≤ 0.05, **p ≤ 0.01, ***p ≤ 0.001. **Abbreviations**: AHBA, Allen Human Brain Atlas; ARCHS4, All RNA‐seq and ChIP‐seq sample and signature search; GO, Gene Ontology; ssGSEA, single‐sample gene set enrichment analysis.

To further characterize the tissue and cell type‐specific expression of ALFF correlated genes, we utilized the All RNA‐seq and ChIP‐seq sample and signature search (ARCHS4) dataset for tissue‐level analysis and the AHBA for cell‐type‐specific characterization. For ALFF positively correlated genes, analysis of the ARCHS4 dataset identified significant enrichment of these genes within specific cortical regions, including the superior frontal gyrus, cerebral cortex, cingulate gyrus, and prefrontal cortex (Figure 4A). At the central nervous system tissue level, the AHBA identified piriform cortex (PCx), olfactory cortex (OCx), dorsal pallium, and the primary somatosensory area as the most enriched regions (Figure 4B). Analysis of the ARCHS4 dataset revealed significant enrichment of these genes within specific tissuesassociated with ECM, including myoblast, adipose tissue, oligodendrocyte, and astrocyte. (Figure S9). At the central nervous system tissue level, the AHBA identified piriform cortex (PCx), stratum oriens, prelimbic area, and the primary somatosensory area as the most enriched regions (Figure S10). The cell‐type specific expression analysis (CSEA) revealed spatiotemporal specificity of ALFF positively correlated genes, with the strongest cortical enrichment observed throughout neurodevelopment. Notably, cerebellar enrichment became evident during critical developmental periods of childhood and adolescent maturation, suggesting temporally regulated involvement of the cerebellum in spontaneous neural activity patterns (Figures S11–S13). While ALFF negatively correlated genes revealed striatum enrichment since late middle fetal and thalamus enrichment during the later stages of neural development. As for cell types, negatively ALFF‐correlated genes were mainly expressed in astrocytes and oligodendrocytes (Figures S14–S16).

Next, we assessed the overlap between ALFF positively correlated genes and marker genes of seven classic neuronal subtypes. The analysis demonstrated a significant association with excitatory neurons (Neuro‐Ex) and inhibitory neurons (Neuro‐In) (Figure 4C). Functional enrichment analysis further indicated that both neuronal subtypes significantly contributed to “chemical synaptic transmission”, “regulation of trans‐synaptic signaling”, and “synaptic membrane organization”. Specifically, Neuro‐Ex was enriched in “neuron‐to‐neuron synapse formation”, “glutamatergic synapse function” and “neuronal cell body pathways”, whereas Neuro‐In was associated with “monoatomic ion channel complexes”, “cognition” and “synapse organization” (Figure 4D). For the overlap between ALFF negatively correlated genes and marker genes of seven classic neuronal subtypes. The analysis demonstrated that negatively ALFF‐correlated genes were mainly overlapped in endothelial cells and glial cells (Figure S17). Functional enrichment analysis further indicated that both endothelial cells and glial cells were enriched in vasculatre development and cell adhesion (Figure S18).

Moreover, ssGSEA characterization of neuronal subtypes revealed distinct spatial enrichment patterns. Neuro‐Ex showed strong cortical enrichment (e.g., superior frontal gyrus: 0.421, inferior parietal cortex: 0.439), which aligns with TED‐associated executive dysfunction and visual processing abnormalities. This cortical enrichment may help explain the observed. ALFF alterations within fronto‐parietal networks. Meanwhile, Neuro‐In displayed preferential associations with subcortical regions (e.g., thalamus: 0.270, amygdala: 0.231), suggesting a potential role of GABAergic modulation within sensory gating and emotional processing circuits (Figure 4E).

### 2.4 PBMC RNA‐Seq Revealed Alterations Consistent with Imaging‐Associated Genes in TED Patients

**FIGURE 5:**
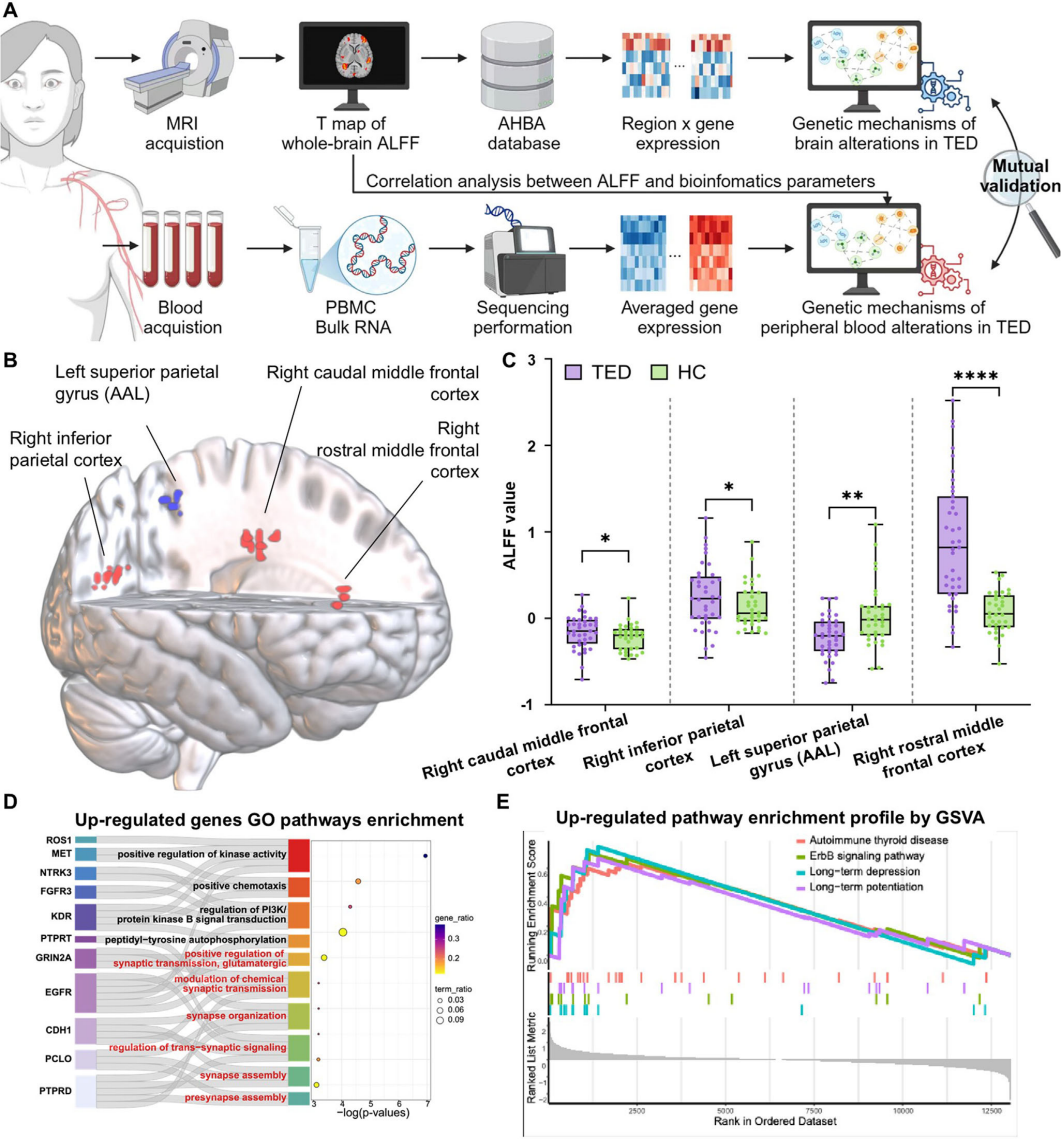
Mutual validation of neural and peripheral immune alterations identifies key pathways in TED pathogenesis. (A) Schematic overview of the multimodal brain–blood validation framework. Imaging–transcriptomic mapping was followed by peripheral PBMC RNA‐seq analysis to identify convergent pathways associated with TED. (B) Significant brain alterations identified by whole‐brain ALFF analysis comparing the TED brain‐blood dataset and the HC brain‐blood dataset. (C) Box plots displaying ALFF values extracted from four significant clusters in TED patients and HCs. The box represents the interquartile range, the line inside the box indicates the median, and the whiskers extend from min to max values, showing the distribution of data. (D) GO pathway enrichment analysis for up‐regulated genes, highlighting the top enriched pathways (adjusted *p* < 0.05). (E) GSVA of up‐regulated pathways in TED patients, highlighting the top four up‐regulated enriched pathways. Significant correlations are marked with asterisks: *p ≤ 0.05, **p ≤ 0.01, ***p ≤ 0.001. Figure 4A was created in BioRender. **Abbreviations**: TED, thyroid eye disease; PBMC, peripheral blood mononuclear cell; ALFF, amplitude of low‐frequency fluctuations; HC, healthy control; AHBA, Allen Human Brain Atlas; RNA‐seq, RNA sequencing; PPI, protein–protein interaction; GO, Gene Ontology; GSVA, gene set variation analysis.

To explore the neural correlates and potential immunological mechanisms underlying TED, we used the TED brain‐blood dataset and HC brain‐blood dataset for further validation of the associated alterations between the brain alterations and peripheral blood transcriptomic changes (Figure 5A). Demographic and clinical profiles of these patients are summarized in Table 1. Whole‐brain ALFF analysis revealed significant differences in the frontal and parietal lobes between TED patients and HCs, consistent with previous identified TED‐related regions from the TED brain test dataset (voxel‐level p < 0.001, cluster‐level p < 0.05, GRF corrected) (Table 2, Figures 5B,C; Figures S19 and S20). For PBMC RNA‐seq, differential expression analysis between TED patients and HCs identified 65 upregulated and 15 downregulated genes (p < 0.05, |logFC| > 1) (Figure S21). PPI network analysis revealed a core module of 10 key genes enriched in cell proliferation (e.g., EGFR, TGFA) and immune regulation (e.g., IL‐10, TGFB2) (Figure S22). Pathway enrichment analysis indicated that upregulated genes were significantly associated with synaptic signaling, including pathways such as “positive regulation of synaptic transmission, glutamatergic”, “modulation of chemical synaptic transmission”, “synapse organization”, “regulation of trans‐synaptic signaling”, “synapse assembly”, and “presynapse assembly”. These findings were consistent with brain imaging data, which also identified positive correlations with genes involved in synaptic function (Figure 5D, Figure S23). KEGG further confirmed associated pathways, including “rap1 signaling pathway”, “calcium signaling pathways”, and “ras signaling pathway” (Figure S24). GSVA confirmed upregulation of neuron activity‐related pathways, such as long‐term potentiation and long‐term depression, as well as pathways of AITD and the ErbB signaling pathway (Figure 5E). Conversely, downregulated pathways were primarily associated with metabolism, including glycosaminoglycan biosynthesis, linoleic acid metabolism, and selenocompound metabolism, along with phototransduction‐related pathways (Figure S25).

**TABLE 2:** Abnormal amplitude of low‐frequency fluctuation in TED brain‐blood dataset (voxel *p* < 0.001, cluster *p* < 0.05, cluster‐level GRF corrected).

Cluster Number	Brain regions (DK atlas)	Brain regions (AAL atlas)	T value	Cluster size	MNI coordinates of peak voxel		
					X	Y	Z
1	Right caudal middle frontal cortex	Right middle frontal gyrus	5.5229	22	36	6	39
2	Right inferior parietal cortex	Right middle frontal gyrus	4.74316	13	51	−51	24
3	Left precuneus cortex	Left superior parietal gyrus	−4.61539	10	−18	−63	48
4	Right rostral middle frontal cortex	Right middle frontal gyrus	4.49218	9	30	51	21
TED: thyroid eye disease; GRF: Gaussian random field; DK: Desikan–Killiany; AAL: automated anatomical labeling; MNI: Montreal Neurological Institute.							

**FIGURE 6:**
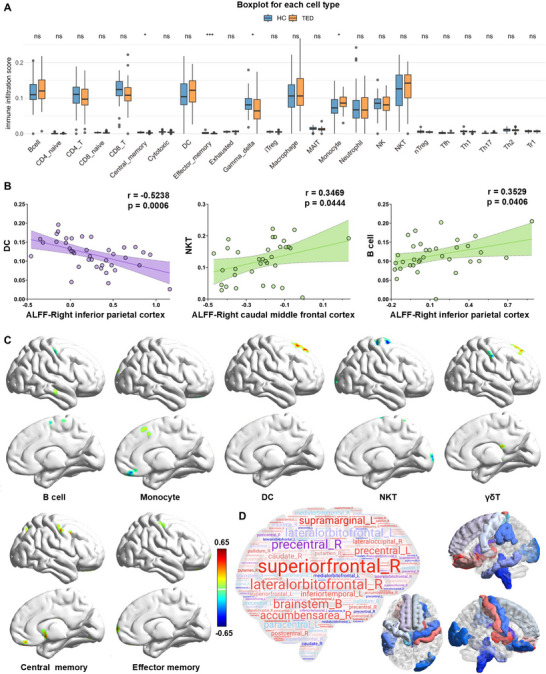
Immune cell infiltration and its association with spontaneous neural activity in TED. (A) Box plots showing immune cell‐type infiltration scores for the *TED brain‐blood dataset* and the *HC brain‐blood dataset*. Significant differences in infiltration between the *TED brain‐blood dataset* and the *HC brain‐blood dataset* are marked with asterisks. (B) Significant correlations between ALFF values from the four significant clusters and immune cell infiltration levels in the *TED brain‐blood dataset* and the *HC brain‐blood dataset*. Purple represents results from TED patients, while green represents results from HCs. The shaded areas indicate the 95% confidence interval. Spearman correlation coefficients (r) and p‐values are displayed for each correlation. (C) Voxel‐wise correlation maps displaying significant associations between whole‐brain ALFF and immune cell infiltration results in TED patients. (D) ROIs mapped onto the DK atlas. Significant clusters identified in (C) were overlaid on the DK atlas, with ROIs defined as parcellations containing ≥6 voxels within a given cluster. The word cloud represents the selected ROIs, with font size proportional to the total voxel coverage of each ROI in (C). The right panel visualizes these ROIs overlaid onto a brain surface representation. Significant correlations are marked with asterisks. * *p* ≤ 0.05, ** *p* ≤ 0.01, *** *p* ≤ 0.001. **Abbreviations**: TED, thyroid eye disease; HC, healthy control; ALFF, amplitude of low‐frequency fluctuations; ROI, region of interest; DK, Desikan–Killiany atlas.

To further explore immune mechanisms potentially underlying these observed transcriptomic changes, we next analyzed immune cell infiltration patterns. This indicated a significant increase in monocyte abundance, alongside a decrease in central memory T cells, effector memory T cells, and γδ T cells in TED patients compared to HCs (Figure 6A; Figure S26). Moreover, in HC, correlation analysis revealed no significant associations among monocytes, central memory T cells, effector memory T cells, and γδ T cells. However, in TED patients, monocytes exhibited significant negative correlations with both central memory T cells and effector memory T cells, while a significant positive correlation was observed between central memory T cells and effector memory T cells (Figures S27 and S28). These findings suggest that immune cell interaction patterns are specifically altered in TED. The observed negative correlations between monocytes and memory T cell subsets may indicate a potential regulatory interplay, whereby increased monocyte abundance is associated with reduced levels of central and effector memory T cells. Meanwhile, the positive correlation between central and effector memory T cells suggests a degree of coordinated regulation within the memory T cell compartment in TED.

### 2.5 Frontoparietal, Subcortical, and Brainstem Areas are Key Brain Regions in TED Brain‐Immune Crosstalk in TED

Given the established alterations in spontaneous neural activity in TED, we next assessed whether these changes are associated with immune infiltration levels in the TED brain‐blood dataset. We specifically focused on peripheral T cell subset infiltration due to the significant altered distribution patterns in TED patients compared to HCs, suggesting its potential role as a pathological biomarker in TED immunopathology.

Analysis of the four differentially expressed ALFF clusters revealed distinct patterns of immune associations in TED patients vs. HCs. In TED patients, ALFF values in the right inferior parietal cortex were negatively correlated with dendritic cell infiltration. In contrast, in HCs, ALFF in the right caudal middle frontal cortex was positively associated with NKT cells, and ALFF in the right inferior parietal cortex was positively correlated with B cells (Figure 6B; Figures S27 and S28).

We further performed voxel‐wise correlation analyses between ALFF values and immune infiltration levels. In TED patients, T cell subset infiltration levels were significantly correlated with ALFF values in widespread brain regions, predominantly localized in the frontal area, with additional involvement of subcortical, parietal, temporal, occipital, insular, and brainstem regions. Notably, the left superior frontal cortex emerged as the region most frequently associated with T cell infiltration patterns in TED patients, whereas HCs exhibited different correlation patterns (Figure 6C; Figures S29 and S30, Table S4).

Similarly, ROI‐based localization from whole‐brain correlation analysis identified 18 TED‐related brain regions significantly correlated with T cell subset infiltration (Figure 6D; Table S5). These regions were distributed across the frontal lobe (e.g., left and right superior frontal cortex, precentral cortex, lateral and medial orbitofrontal cortex), parietal lobe (e.g., supramarginal cortex, inferior temporal cortex), occipital lobe (e.g., right lateral occipital cortex), subcortical structures (e.g., caudate, putamen, pallidum, accumbens area), and brainstem. Notably, several regions, including the superior frontal cortex (bilateral), postcentral cortex (right), precentral cortex (bilateral), and brainstem, demonstrated consistent associations across both the TED brain test dataset and the TED brain‐blood dataset.

Collectively, the observed ALFF‐immune correlations concentrated in the frontal lobe, subcortical structures, parietal lobe, and brainstem. These brain‐immune correlations were consistently observed in both TED cohorts, highlighting the potential role of neuroimmune interactions in TED pathogenesis, which gives a deeper insight into the brain‐immune crosstalk in AITD.

## 3 Discussion

As the representative type of AITD, TED impairs patients’ visual function and appearance, yet accumulating evidence has shown that its pathogenic influence extends to the brain, suggesting potential complex brain‐immune interactions [17]. By integrating neuroimaging with transcriptomic analysis, this study preliminarily explored the regional functional signatures and potential brain‐immune crosstalk underlying TED. The retrospective cohort revealed disease‐related alterations in spontaneous neural activity, primarily involving frontal, parietal, subcortical regions, and brainstem, and these findings were validated in a separate prospective cohort. Crucially, the observed functional alterations were intimately associated with transcriptional signatures implicated in vascular processes, synaptic signaling pathway, light response, metabolic activity, and immune status. The positive‐related genes exhibited enrichment in excitatory and inhibitory neurons, which was also distributed in a unique pattern in the brain, thereby providing molecular insights into brain‐immune crosstalk in TED as well as AITD. The significant associations between immune infiltration levels and neural activity patterns further underscored the pivotal role of systemic immune response and facilitated the validation of brain‐immune crosstalk in AITD.

Through ALFF‐clinical correlation analysis in the TED brain test dataset, it was preliminarily identified that frontal, parietal, subcortical regions, and brainstem served as potential disease‐related brain regions in TED. Notably, alterations in these regions were further confirmed in the prospective cohort, and these regions also emerged as key nodes associated with peripheral immune infiltration patterns. The spatial convergence between ALFF–clinical and ALFF–immune correlation‐defined ROIs suggests that these regions may represent shared neurobiological substrates integrating functional, endocrine, and immune alterations in TED [12, 18]. From a biological perspective, the frontal lobe plays a pivotal role in oculomotor function, cognition, and emotion, and the parietal lobe is implicated in linking sensory processing with behavioral decision [19, 20]. Besides, previous studies have revealed the impact of peripheral inflammation on frontal, parietal, subcortical regions, and the brainstem, which may further lead to neuropsychiatric abnormalities [21, 22]. Thus, abnormal spontaneous functional activity in these brain regions might not only suggest the visual dysfunction and higher‐order function disorder in TED, but also indicate the neuroinflammatory mechanism in AITD. However, there is great heterogeneity of TED and its concomitant symptoms, resulting in extensive and non‐specific brain alterations [23, 24]. Importantly, while TED serves as a representative and clinically accessible model, these findings should primarily be interpreted as TED‐related neuroimmune alterations, with potential relevance—but not direct equivalence—to other AITD subtypes. Though the frontal and parietal regions were ascertained as key regions in an abundant of studies [25], further image‐related gene analysis is still required to validate the neuroimaging discovery and explore the peripheral inflammation impact in AITD. At the same time, we acknowledge that ALFF is a sensitive index of spontaneous neural activity and may exhibit methodological convergence across different correlation analyses. The overlap between ALFF–clinical and ALFF–immune ROIs may therefore partly reflect the intrinsic sensitivity of ALFF to disease‐related neural perturbations. However, the reproducibility of these spatial patterns across independent cohorts, combined with their biological plausibility and convergence with immune associations, argues against a purely methodological explanation. Instead, these findings highlight frontal, parietal, subcortical, and brainstem regions as key vulerable regions of brain–immune crosstalk in TED, warranting further validation through longitudinal and mechanistic studies.

The ALFF‐related transcription alterations in TED were mainly found in genes associated with vascular process, synaptic signaling, light response, metabolic activity, and immune response. Previous studies uncovered increased orbital vascular development induced by a microenvironment with rich vascular endothelial growth factor, and TED patients may suffer orbital edema due to characteristics of immature blood vessels [26]. Increased fenestrated vessels were also revealed in autoimmune thyroid tissue in AITD, which may result from abnormal gene transcriptions related with angiogenesis and permeability [27]. The brain enrichment pattern of vascular‐related genes may suggest the association between subcortical regions and brain activity, and helps elucidate the vascular‐related mechanism in AITD. The synapses are the basic unit converting electric signals to chemical molecules for information transmission. Dysfunction of the synapse is recognized as hallmarks of neurodegenerative diseases, which may be caused by a genetic disorder or an autoimmune attack [28, 29]. The synaptic dysfunction was demonstrated in AITD, reflecting neural microstructure lesions [30]. Notably, thyroid disorders may exert an impact on the systemic metabolic status as well as brain functional activity [31]. As a critical regulator of metabolism and neural development, thyroid hormones act on structure and activities in multiple brain areas, and may play a role in neural protection [32, 33]. More interestingly, retinal cells modulate the light/dark adaptation via thyroid hormone signaling [34]. The positively correlated genes regarding synapse signaling and light response may indicate the neural impairment and compensatory process, and highlight the impact of thyroid hormones, giving more clues about neural changes in TED. In addition, consistent with gene transcription signatures, AITD is featured with autoimmune reaction, especially lymphocyte infiltration and autoantibody attack [35]. More specifically, TED demonstrated an abnormal local orbital immune microenvironment, with higher infiltration levels of T cells and cytokines [36]. The orbital fibroblasts express thyroid‐stimulating hormone receptor and insulin‐like growth factor‐1 receptor, which may be cross‐linked by autoimmune antibodies and induce inflammatory reaction, fibrosis, and adipogenesis processes [37]. Therefore, gene transcription signatures related to brain activities facilitate a comprehensive understanding of the TED mechanism and validate disease‐related brain alterations.

The subsequent tissue‐level and cell‐type‐specific analysis revealed that positive‐related genes displayed enrichment in Neuro‐Ex and Neuro‐In in the brain tissue. These two neuronal subtypes release excitatory or inhibitory neurotransmitters to regulate the synaptic signaling pathway, and there exists a fine balance between the activities of Neuro‐Ex and Neuro‐In [38]. Abnormalities of Neuro‐Ex or Neuro‐In may suggest a neurological disorder. Besides, ssGSEA analysis unraveled the enrichment of Neuro‐Ex in cortical regions such as the frontal and parietal cortex, consistent with brain activity alterations. The hyperactivity of synapse‐related genes in Neuro‐Ex may help elucidate the ALFF alterations. Meanwhile, the related Neuro‐In is mainly located in subcortical regions, which communicate with multiple cortical regions to regulate social cognition and behavior [39]. The alterations in Neuro‐In transcription may explain an impaired functional network integration in TED.

The unique immune landscape in TED was depicted through immune infiltration analysis in the brain‐blood dataset, in which the T cell subsets and monocytes exhibited significant differences. Notably, peripheral immune cells may serve as indirect indicators of autoimmune attack against the thyroid and orbit [40]. Previous studies have shown that immune cell infiltration within the thyroid gland contributes to thyroidal destruction in AITD, emphasizing the broader systemic immune dysregulation [41]. Similarly, orbital infiltrated T cells and monocytes may modulate the fibrosis and inflammatory reaction in TED [42, 43]. Given that the eyes and the brain are interconnected in immune reaction, the autoimmune responses targeting the orbit may subsequently modulate brain function through localized orbital inflammation [16]. Moreover, peripheral immune cells can exert an impact on the brain without direct infiltration into the brain, suggesting a systemic inflammatory route linking peripheral immunity to central neural alterations [44]. Hence, these immune alterations likely represent autoimmune inflammation extending beyond local orbital and thyroidal tissues, potentially influencing central neural circuits via systemic inflammatory mediators. The observed abnormal immune cell population thus provides additional validation of the immune mechanism underlying TED, and significant ALFF‐immune correlations provide a deeper insight into the brain‐immune crosstalk.

Though this study delineated transcriptional signatures associated with regional brain activity alterations and revealed coordinated patterns between central neural function and peripheral immune status in TED, several limitations should be acknowledged. First, while the key frontoparietal alterations were consistently identified across two independent datasets, the relatively modest sample size of the prospective cohort may limit the statistical power for detecting subtle neuroimmune interactions. Consequently, while the core findings are robust, the more intricate associations between specific immune cell subsets and brain activity should be considered preliminary and exploratory, requiring further validation in larger, multi‐center cohorts. Second, future investigations utilizing high‐field MRI, dedicated processing pipelines, and specialized parcellation techniques will be essential to delineate the involvement of discrete nuclei and to mitigate potential physiological artifacts. Specifically, the lack of direct blood‐brain barrier (BBB) permeability assessment means that we cannot definitively determine the structural integrity of the barrier in our patients. Future studies incorporating markers of endothelial dysfunction and BBB integrity are needed to elucidate the exact physical pathways of the observed brain‐immune crosstalk. Additionally, since the gene transcription signatures are based on the AHBA atlas and not rs‐fMRI subject brain tissue, they lack individual correspondence. Accordingly, the observed imaging–transcriptomic associations should be interpreted as spatial correspondences between regional brain functional alterations and conserved transcriptional patterns, rather than as direct, real‐time, or causal molecular changes occurring in TED patients. Furthermore, while peripheral blood transcriptomics reflects systemic gene expression, ethical constraints precluded the use of orbital tissue or cerebrospinal fluid for more direct transcriptional and proteomic profiling. Future animal studies may address this limitation. Importantly, although significant correlations were observed between brain functional alterations and peripheral immune transcriptional signatures, these findings should be interpreted as association‐based rather than mechanistic. The cross‐sectional, multimodal correlation framework does not allow causal inference regarding the directionality or biological drivers of the observed brain–immune coupling. Instead, our results define a systems‐level pattern of coordinated neuroimmune alterations in AITD, serving as a hypothesis‐generating foundation for future longitudinal and mechanistic investigations.

## 4 Conclusion

In conclusion, by decoding transcriptomic and neuroimaging signatures, this study underscores the intricate brain‐immune crosstalk inherent to TED pathogenesis. The frontal, parietal, subcortical regions, and brainstem were identified as key disease‐related regions. The underlying transcription signatures were further unraveled through AHBA and peripheral transcriptomics, which validated the altered transcription of genes associated with synaptic signaling, neurovascular regulation, and immune activation. The integration of brain‐wide transcriptional and peripheral immune profiles has advanced our understanding of the molecular underpinnings linking autoimmune inflammation with neural alterations in TED.

## 5 Experimental Section

### 5.1 Participants

This study was approved by the Ethics Committee and was in compliance with the Declaration of Helsinki. Written informed consent was obtained from all participants. The study included two distinct TED patient cohorts: a retrospective cohort aimed at identifying TED‐related brain areas by correlating rs‐fMRI data with clinical indicators, and exploring associated genetic pathways; and a prospective cohort designed to validate these brain regions and genetic mechanisms by comparing TED patients to HCs, integrating peripheral blood RNA‐sequencing data. Detailed information on participants’ inclusion and exclusion process was displayed in Figure S1.

#### 5.1.1 Retrospective Cohort (TED Brain Test Dataset)

In the retrospective part of our study, we retrospectively recruited 155 TED patients who underwent rs‐fMRI at Shanghai Ninth People's Hospital between December 2022 and May 2024. TED diagnosis was based on the clinical guidelines proposed by the European Group on Graves’ Orbitopathy (EUGOGO) [45]. Disease duration was determined from the onset of ocular manifestations. TED clinical activity was assessed using the clinical activity score (CAS), supplemented by orbital MRI findings [46]. All eye examinations were performed by the same certificated person at the hospital. The serum thyroid function tests and thyroid antibody tests were performed by the clinical laboratory.

Exclusion criteria included (1) history or clinical evidence of ophthalmic diseases unrelated to TED; (2) prior ocular surgery; (3) psychiatric or neurological disorders; (4) concurrent endocrine diseases other than thyroid dysfunction; (5) anatomical brain abnormalities; (6) inadequate MRI image quality compromising analytical accuracy; and (7) signs indicative of dysthyroid optic neuropathy [18]. After applying these criteria, 116 TED patients remained eligible for inclusion and served as the TED brain test dataset.

#### 5.1.2 Prospective Cohort (TED Brain‐Blood Dataset, HC Brain‐Blood Dataset)

To validate the findings from the TED brain test dataset, we prospectively recruited a cohort comprising 39 TED patients from September 2024 to November 2024 as the TED brain‐blood dataset. In addition to undergoing rs‐fMRI, the prospective cohort underwent peripheral blood collection, PBMC isolation, and bulk RNA‐Seq analysis, enabling a focused investigation of immune‐related gene signatures. Additionally, 42 age‐, and sex‐ matched HCs were enrolled for comparative analyses as the HC brain‐blood dataset. Clinical indicators were collected in the same way as before.

### 5.2 MRI Acquisition and Data Processing

MRI acquisition and data processing were the same as our previous study [18]. Wakeful rs‐fMRI was performed on a 3T scanner (Magnetom Vida, Siemens) using a 64‐channel phased‐array head coil. The entire brain was covered by high‐resolution sagittal structural T1‐weighted images and functional images. Participants were instructed to close their eyes and remain awake. Head motion and noise were minimized using foam padding and earplugs.

rs‐fMRI data was conducted using DPABI version 7.0 (http://rfmri.org/DPABI) on MATLAB (www.mathworks.com/products/matlab) [47]. Preprocessing included discarding the first 10 volumes, slice timing correction, realignment, normalization to MNI space (resampled to 3‐mm isotropic voxels), spatial smoothing (6‐mm full‐width at half‐maximum Gaussian kernel), detrending, and nuisance covariate regression (cerebrospinal fluid, white matter signals, and head motion parameters).

The amplitude of low‐frequency fluctuation (ALFF) is a promising and frequently used method for detecting the regional intensity of spontaneous blood‐oxygen‐level‐dependent signal with a clear neural basis [48]. Thus, we computed ALFF across the whole brain by transforming voxel time‐series data into the frequency domain (0.01–0.08 Hz) using Fast Fourier Transform. ALFF values were standardized by dividing by the global mean ALFF and normalized with Fisher's z‐transformation. MRI sequence parameters and detailed data processing descriptions are provided in Supplementary Text.

### 5.3 ALFF Spatial Correlation to TED Clinical Indicators

To investigate the relationship between whole‐brain ALFF and TED‐related clinical indicators (disease duration, CAS, proptosis, TSH, fT3, fT4, and TRAb), voxel‐wise correlation analyses were performed across the TED brain test dataset. Multiple comparisons were corrected using GRF correction, with a voxel‐level threshold of *p* < 0.005 and a cluster‐level threshold of *p* < 0.05 (two‐tailed).

To define functionally relevant anatomical regions for subsequent image‐transcriptomic integration, significant clusters were mapped onto the DK atlas. We implemented a minimum spatial extent criterion, defining an ROI as any DK parcel containing at least 6 significant voxels. This threshold was specifically chosen to exclude small, potentially spurious clusters and ensure that each ROI represents a robust, spatially meaningful intersection between anatomical boundaries and disease‐related functional alterations. Finally, 19 ROIs were identified.

### 5.4 Brain Transcriptional Data Processing

Regional microarray expression data were obtained from 6 post‐mortem brains provided by the Allen Human Brain Atlas (AHBA, https://human.brain‐map.org) [49]. Data processing was conducted using the abagen toolbox (version 0.1.3; https://github.com/rmarkello/abagen) with an 83‐region defined as the DK atlas in MNI space. Microarray probes were reannotated following a previous study [50]. Unreliable probes (those lacking valid Entrez IDs or exhibiting low expression intensity in ≥50% samples) were removed [51]. When multiple probes corresponded to the same gene, the probe with the most stable regional variation across donors was selected using differential stability. Tissue samples were spatially aligned to the DK atlas in MNI space and assigned to specific regions if located within 2 mm of atlas parcels, constrained by hemisphere and structural divisions. Samples not assigned to any region in the provided atlas were excluded. To correct inter‐subject variability, gene expression values were normalized across samples using a robust sigmoid normalization followed by rescaling to the unit interval. Finally, samples assigned to the same brain region were averaged first within each donor, then across donors, generating a standardized regional gene expression matrix.

Gene expression data were extracted from 83 brain regions from the DK atlas, and 19 ROIs were identified from correlation analyses in the TED brain test dataset. Due to data limitations, one ROI (right temporal pole) had no expression data and was thus excluded from subsequent analyses. Regional gene expression was then z‐score standardized for subsequent analysis. Full methodological details and equations are provided in Supplementary Text.

### 5.5 ALFF Spatial Correlation to AHBA Transcriptional Profiles

We harmonized the spatial reference between ALFF maps and AHBA transcriptional profiles by parcellating both modalities with the same DK atlas. The DK atlas was resampled to each participant's ALFF image. For each of the 83 DK regions, we computed the mean ALFF value. We next investigated whether transcriptional profiles derived from AHBA were associated with whole brain ALFF map of the TED brain test dataset. Pearson correlation coefficients were computed between ALFF values and the expression levels of each gene across all 82 cortical regions and all 19 ROIs. To control for multiple comparisons, FDR correction was applied using the Benjamini–Hochberg method. Genes with FDR‐corrected p < 0.05 were considered significantly correlated with ALFF.

### 5.6 Image‐Related Gene Network Analysis with Tissue‐Cell Specific Functional Mapping

To explore the functional features of image‐related genes, we conducted protein PPI and enrichment analyses. The PPI network of the significantly related genes was constructed using the STRING database (https://string‐db.org/). Subsequently, the PPI network underwent analysis through the molecular complex detection (MCODE) algorithm in Cytoscape [52, 53]. The following parameters were applied: degree cutoff = 2, node score cutoff = 0.2, k‐core = 2, and maximum depth = 100, which are commonly used to balance module sensitivity and specificity. GO analyses and KEGG analyses were conducted using the clusterProfiler R package (version 4.10.0) and GseaVis (https://github.com/junjunlab/GseaVis), with gene set enrichment analysis (GSEA) also performed on the image‐related genes based on their correlation [54]. The pathway networks were constructed by Metascape (https://metascape.org/) [55]. Then, the ssGSEA algorithm was performed to identify ssGSEA scores as quantificationally interested pathway related levels in each brain region with the ‘Gaussian’ regression and ‘gsva’ algorithm by the GSVA package [56].

To assign image‐related genes to specific tissue and cell types, we utilized Enrichr (https://www.maayanlab.cloud/Enrichr/) to perform analyses on positive‐related genes. The ARCHS4 dataset was employed for tissue type determination, while the AHBA was used for cell type assignment [57]. We also utilized the online CSEA tool (http://genetics.wustl.edu/jdlab/csea‐tool‐2/) for further validation [58]. Then, the marker gene lists of seven classical brain‐cell types (microglia, oligodendrocyte precursors, endothelial cells, oligodendrocytes, astrocytes, inhibitory and excitatory neurons) were obtained following the procedure in the study of Seidlitz et al. [59]. We overlapped the positive‐related genes with marker genes of these cells and conducted functional enrichment analysis using the clusterProfiler R package based on the overlapped genes in each cell type [60]. Subsequently, we quantified cellular related levels in each brain region using the ssGSEA algorithm [56].

### 5.7 Mapping of ALFF Signatures to Pathways and Cell Types

To investigate the association between spontaneous neural activity and transcriptional signatures, we performed voxel‐wise correlation analyses between whole‐brain ALFF maps and selected pathways and seven brain cell types as determined by the ssGSEA algorithm across the TED brain‐blood dataset and HC brain‐blood dataset. The immune cell types were computed using ssGSEA with an immune‐related gene applied to PBMC bulk RNA‐seq data. Subsequent correlation analyses were conducted between these scores and whole‐brain ALFF maps, aiming to explore relationships between peripheral immune status and brain alterations in TED. Multiple comparisons were corrected using GRF correction, with a voxel‐level threshold of p < 0.005 and a cluster‐level threshold of *p* < 0.05 (two‐tailed).

### 5.8 Peripheral Blood Profiling and RNA Sequencing Validation

The TED brain‐blood dataset and the HC brain‐blood dataset were collected from 39 TED patients and 34 HCs in EDTA anticoagulant tubes and stored at 4°C for immediate processing. PBMCs were isolated via Ficoll density gradient centrifugation. Briefly, whole blood was diluted 1:1 with PBS, layered over Ficoll solution, and centrifuged at 400 g for 30 min (room temperature, acceleration/brake set to 1). The PBMC layer was collected, washed with PBS (500 g, 10 min), and pelleted. Samples with erythrocyte contamination were treated with ACK Lysing Buffer (Gibco, A10492‐01) for analysis.

Total RNA was extracted using the Total RNA Extractor (Sangon, B511311). Libraries were sequenced on a DNBSEQ‐T7 platform. Raw reads were processed with fastp (v0.12.4), aligned to GRCh38 via HISAT2 (v2.2.0), and transcript abundance was quantified as raw counts using StringTie (v2.2.1) [61, 62, 63]. Using the R package Limma (version 3.58.1) for raw count data normalization and differential expression analysis [64]. As a result, we obtained a log count per million differentiation index per gene, where positive values indicate higher expression in TED patients compared to HCs. We constructed a PPI network on all differential expressed genes and pathway enrichment on up‐regulated expressed genes as described before. These enriched pathways were subsequently compared with those previously identified from image‐related genes to identify shared biological mechanisms. The immune cell‐type score of each sample was calculated using the ssGSEA method based on gene signatures of the 24 immune cell‐types from ImmuneCellAI [65].

### 5.9 Statistical Analysis

Data normality was tested using the D'Agostino‐ Pearson omnibus normality test. For normally distributed data, statistical significance was assessed using independent‐sample t tests; for non‐normally distributed data, Mann–Whitney U tests were applied. A *p* <0.05 indicated a statistically significant difference.

The DPABI toolbox 7.0 (http://rfmri.org/DPABI) was used for analyzing ALFF between TED and HCs [47]. Multiple comparison correction was conducted within the whole brain, and significant clusters were identified using a voxel‐level threshold of p <0.001 and a cluster‐level threshold of p <0.05 (GRF correction) with a two‐tailed test. MRIcroGL version 1.2.20220720 (https://www.nitrc.org/projects/mricrogl) and BrainNet Viewer version1.7 (https://www.nitrc.org/projects/bnv) were used to visualize group differences and correlation results [66, 67]. For bioinformatics analysis, R (version 4.4.3) were used. Data visualization was performed using ggplot2 (version 3.5.1) and tidyplots (version 0.2.2.9000) [68, 69].

## Funding

National Natural Science Foundation of China (82388101, 82071003 and 82271122), Shanghai Eye Disease Research Center (2022ZZ01003), Biobank Project of Shanghai Ninth People's Hospital (YBKB202211), Shanghai Municipal Commission of Health and Family Planning Project (2022XD006), Research Center for Eye Disease and Visual Rehabilitation, and the Key Project of Yuanshen Rehabilitation Institute, Shanghai Jiao Tong University School of Medicine (yskf1‐24‐0926‐2, yskf2‐24‐0926‐2).

## Conflicts of Interest

The authors declare no conflicts of interest.

## Supporting information




**Supporting File**: advs74686‐sup‐0001‐SuppMat.pdf.

## Data Availability

The data that support the findings of this study are available from the corresponding author upon reasonable request.
