# Supplementary material for: Transcriptomic and Neuroimaging Decoding of Brain‐Immune Crosstalk in Thyroid Eye Disease
Source: Adv Sci (Weinh). 2026 Mar 5;13(27):e23609. doi: 10.1002/advs.202523609 (PMC13170231; doi:10.1002/advs.202523609)
Supplement: Supplementary file 1 — Supporting File: advs74686‐sup‐0001‐SuppMat.pdf. [file ADVS-13-e23609-s001.pdf]

Supporting Information

Transcriptomic and neuroimaging decoding of brain-immune crosstalk in thyroid eye disease

Haiyang Zhang, Yuting Liu, Shufan Jiang, Zilin Fang, Mengda Jiang, Xiaofeng Tao, Tianyi Zhu, Jipeng Li, Sijie Fang, Xuefei Song, Yinwei Li, Jing Sun, Chuanjun Tong, Zhengrun Gao, Huifang Zhou, Xianqun Fan

Supplementary Methods

MRI acquisition and data processing

MRI acquisition and data processing were the same as our previous study[1]. Wakeful resting-state functional magnetic resonance imaging (rs-fMRI) was performed on a 3T scanner (Magnetom Vida, Siemens) using a 64-channel phased-array head coil. The entire brain was covered by high-resolution sagittal structural T1-weighted images and functional images. To minimize head motion and scanning noise, foam padding and earplugs were used. During scanning, all participants were instructed to close their eyes while remaining awake, thereby reducing vigilance-related interference[2]. The high-resolution sagittal structural T1-weighted images (3D-T1WI) for rs-fMRI, with the following parameters (TR = 2,400 ms, TE = 2.4 ms, thickness = 0.8 mm, gap = 0 mm, matrix = 320 × 320, FOV = 256 × 256 mm^2, FA = 8°, number of slices = 208 and voxel size = 0.8 × 0.8 × 0.8 mm^3) and functional images with the parameters (TR = 2000 ms, TE = 30 ms, thickness = 2 mm, matrix = 104 ×104, FOV = 208 × 208 mm^2, FA = 90°, number of slices = 72, gap = 0 mm, and voxel size = 2 × 2 × 2 mm^3) were obtained.

Rs-fMRI data was conducted using DPABI version 7.0 (http://rfmri.org/DPABI) on the MATLAB platform (www.mathworks.com/products/matlab)[3]. The first 10 functional volumes were eliminated for magnetization balancing. Slice timing and realignment for head motion correction were performed. Images were then normalized to the Montreal Neurological Institute (MNI) standard space using a linear registration algorithm and resampled to a voxel size of 3 mm × 3 mm × 3 mm. Spatial smoothing was applied using a 6-mm full-width at half-maximum Gaussian kernel. To remove linear trends, detrending was conducted. Additionally, nuisance covariates were regressed out, including average signals from cerebrospinal fluid and white matter, as well as six head motion parameters. Participants were excluded from the final analysis if the maximal translational or rotational head movement exceeded 3.0 mm or 3.0°.

The amplitude of low-frequency fluctuation (ALFF) is a promising and frequently used method for detecting the regional intensity of spontaneous blood-oxygen-level-dependent signal with clear neural basis [4]. Thus, we computed ALFF across the whole brain. Time courses data were converted to the frequency domain with a fast Fourier transform algorithm to enable each voxel to contain the amplitude of the signal across the whole spectrum. The averaged square root of the spectrum spanning the frequency range of 0.01-0.08 Hz was taken as the ALFF measurement. For standardized variability among the participants, the mean ALFF was obtained as the ALFF value divided by the global mean ALFF value. Fisher's z transformation was then performed for normalization and statistical analysis facilitation.

Brain transcriptional data processing

Regional microarray expression data were obtained from 6 post-mortem brains provided by the Allen Human Brain Atlas (AHBA, https://human.brain-map.org)[5]. Data processing was conducted using the abagen toolbox (version 0.1.3; https://github.com/rmarkello/abagen) with an 83-region defined as the Desikan-Killiany (DK) atlas in MNI space.

Microarray probes were reannotated following a previous study[6]. Probes without a valid Entrez ID were discarded. To filter out unreliable probes, expression intensity was compared to background noise, and probes were removed if their intensity was below background levels in ≥50% of samples across donors[7]. When multiple probes corresponded to the same gene, the probe with the most stable regional variation across donors was selected using differential stability, defined as[8]:

$$\Delta_S(p) = \frac{1}{\binom{N}{2}} \sum_{i=1}^{N-1} \sum_{j=i+1}^{N} \rho\left[B_i(p), B_j(p)\right]$$

where ρ is Spearman's rank correlation of the expression of a single probe *p* across regions between two donors B_i and B_j, and *N* is the total number of donors. Brain regions were defined according to the structural ontology provided by the AHBA.

The MNI coordinates of tissue samples were updated to those generated via non-linear registration using the Advanced Normalization Tools (ANTs; https://github.com/chrisfilo/alleninf). Samples were assigned to brain regions in the provided atlas if their MNI coordinates were within 2 mm of a given parcel. To minimize misassignment, sample-to-region matching was constrained by hemisphere and gross structural divisions (i.e., cortex, subcortex/brainstem, and cerebellum). For example, a sample in the left cortex could only be assigned to an atlas parcel in the left cortex[6]). Samples not assigned to any region in the provided atlas were excluded.

Inter-subject variation was addressed by normalizing tissue sample expression values across genes using a robust sigmoid function[9]:

$$x_{norm} = \frac{1}{1 + \exp\left(-\frac{(x - \langle x \rangle)}{\mathrm{IQR}_x}\right)}$$

where $\langle x \rangle$ is the median and IQR_x is the normalized interquartile range of the expression of a single tissue sample across genes. Normalized expression values were further rescaled to the unit interval:

$$x_{scaled} = \frac{x_{norm} - min(x_{norm})}{max(x_{norm}) - min(x_{norm})}$$

Finally, gene expression values were then normalized across tissue samples using the same procedure. Expression values for samples assigned to the same brain region were averaged first within each donor and then across donors, producing a regional gene expression matrix.

Gene expression data were extracted from 83 brain regions from DK atlas and 19 regions of interest (ROIs) identified from correlation analyses in all TED patients using the processed AHBA data. Due to data limitations, one ROI, the right temporal pole, had no expression data and was thus excluded from subsequent analyses. To enable cross-region comparisons, the expression data were z-score normalized across all cortical regions, resulting in a final 82 ×15633 (regions × genes) matrix and a final 19 ×15633 (regions × genes) matrix that captured standardized gene expression patterns for further analysis.

Supplementary Results

Association between spontaneous neural activity and clinical indicators in TED brain test dataset

CAS was positively associated with ALFF in subcortical structures, notably the left thalamus, but negatively associated with ALFF in cortical regions, including the left inferior temporal, superior frontal, and supramarginal cortices. Disease duration showed extensive positive correlations across subcortical (brainstem, thalamus, amygdala, hippocampus, caudate) and cortical regions (bilateral insular cortex, fusiform and middle temporal cortices, entorhinal cortex, and right supramarginal cortex). Proptosis demonstrated positive ALFF correlations mainly in frontal-parietal areas (right rostral and caudal middle frontal cortices, precentral gyrus, supramarginal cortex), whereas a negative correlation appeared in the left superior temporal cortex.

Serum thyroid function and auto-antibody parameters also exhibited significant spatial associations with ALFF. Serum TSH correlated positively with ALFF predominantly within bilateral frontal regions (pars orbitalis, rostral anterior cingulate, superior frontal, and rostral middle frontal cortices), extending to right inferior parietal and superior temporal cortices, whereas a negative correlation was limited to the left fusiform cortex. Elevated serum fT3 levels corresponded to widespread increases in ALFF across frontal, parietal, temporal, and subcortical regions (particularly rostral middle frontal, superior frontal, precentral, postcentral, supramarginal cortices, and caudate nucleus). Similarly, higher serum fT4 correlated positively with ALFF, specifically in the right rostral middle frontal, superior frontal, left superior temporal cortices, and the left thalamus. In addition, elevated TRAb levels correlated positively with ALFF, especially in bilateral superior parietal, right inferior parietal, superior-middle temporal, and lateral occipital cortices.

Altered spontaneous neural activity in frontal and parietal regions of TED brain-blood dataset

Whole-brain ALFF analysis revealed four brain regions with significant differences between *TED brain-blood dataset* and *HC brain-blood dataset*, including increased ALFF in the right caudal middle frontal cortex, right inferior parietal cortex, and right rostral middle frontal cortex, and decreased ALFF in the left precuneus cortex (voxel-level $p < 0.001$, cluster-level $p < 0.05$, GRF corrected; Figure 5B, Figure S19, Supporting information). These regions are primarily located in the frontal and parietal lobes, consistent with the TED-related regions previously identified in the *TED brain dataset*. Notably, the right caudal middle frontal cortex, the right inferior parietal cortex and right rostral middle frontal cortex overlapped with regions implicated in the earlier ALFF-clinical correlation analysis. Moreover, in the *TED brain-blood dataset*, voxel-wise correlation analyses between ALFF and clinical indicators showed spatial patterns highly similar to those observed in the *TED brain dataset* (Figure S20, Supporting information), reinforcing the robustness of the disease-associated neural activity alterations.

Convergent frontoparietal and subcortical brain-immune associations characterize TED patients

Given the established alterations in spontaneous neural activity in TED, we further performed voxel-wise correlation analyses between ALFF values and immune infiltration levels in the *TED brain-blood dataset* and *HC brain-blood dataset*.

Specifically, B cell infiltration was significantly correlated with ALFF values in regions predominantly located in the frontal and temporal lobes in TED. Negative correlations were observed in the left superior frontal cortex, bilateral precentral cortices, and the left paracentral lobule, all within the frontal lobe. In contrast, positive correlations were detected in the left lateral orbitofrontal cortex, the right middle temporal cortex, and the right superior frontal cortex.

For monocyte infiltration, negative correlations were observed in the right lateral orbitofrontal cortex, the left inferior temporal cortex, and the left lateral orbitofrontal cortex, involving both the frontal and

temporal lobes. Positive correlations were found in the right superior frontal cortex and the right lateral occipital cortex, spanning the frontal and occipital regions.

DC infiltration demonstrated extensive negative correlations with ALFF in widespread areas, including the left supramarginal gyrus (parietal lobe), right insular cortex, bilateral fusiform gyri (temporal lobe), the right rostral middle frontal cortex (frontal lobe), and multiple subcortical regions (including the accumbens area, hippocampus, pallidum, putamen, caudate, and thalamus). Positive correlations were found in the bilateral postcentral gyri, located in the parietal lobe.

For NKT cells, significant negative correlations were predominantly identified in frontal and occipital regions, including the right superior frontal cortex, right precentral cortex, right paracentral lobule, and occipital areas such as the pericalcarine, lingual, and lateral occipital cortices.

Regarding γδ T cell infiltration, positive correlations were primarily localized to the parietal and frontal lobes, as well as subcortical areas. Specifically, positive associations were observed in the left inferior parietal cortex, right superior frontal cortex, right postcentral cortex, and the brainstem. Negative correlations were noted in the right postcentral cortex and left precentral cortex.

For central memory T cells, positive correlations with ALFF were widespread across multiple regions, including the bilateral lateral orbitofrontal cortices, right precentral cortex, caudal middle frontal cortex, and subcortical areas such as the caudate and accumbens. Additionally, associations were observed in the right superior parietal cortex and the right lingual cortex.

Lastly, effector memory T cell infiltration showed significant positive correlations with ALFF in regions restricted to the frontal lobe, including the right superior frontal cortex, right lateral orbitofrontal cortex, and the left medial orbitofrontal cortex.

Supplementary Figures S1–S30:

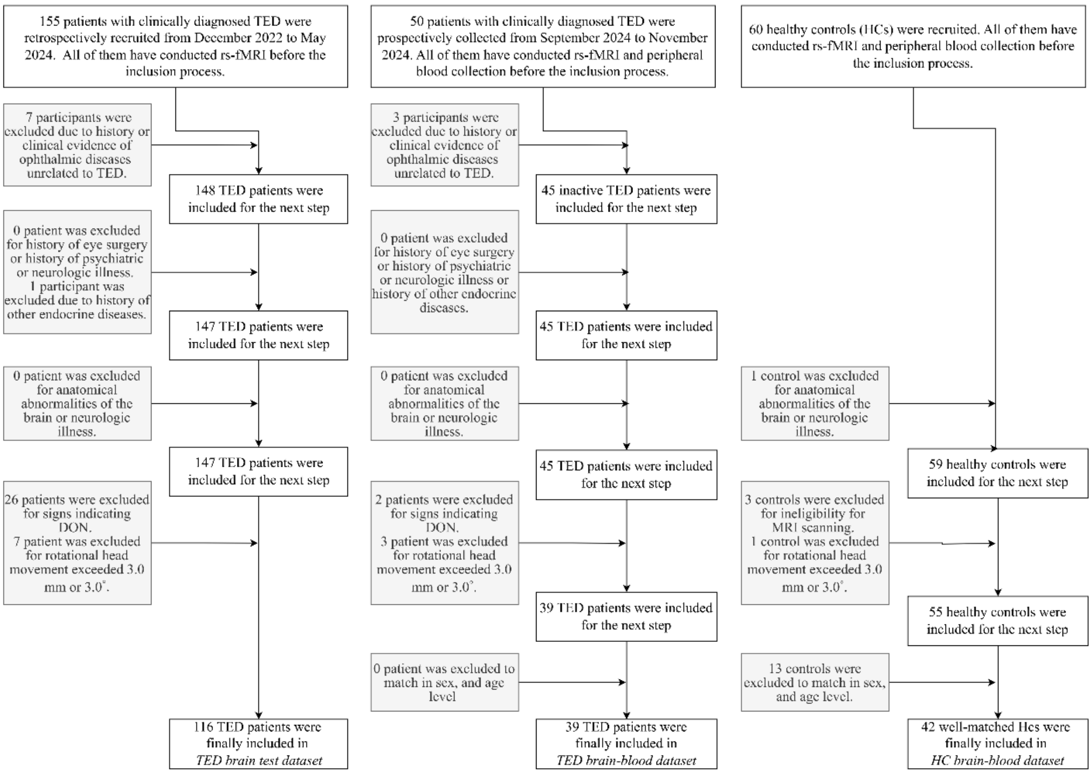

Figure S1. Flowchart of Participant Recruitment and Screening for the Study Cohorts.

A total of 155 TED patients were retrospectively recruited, with 116 patients meeting inclusion criteria and included in the *TED brain test dataset*. A separate prospective cohort of 50 TED patients was recruited, resulting in 39 patients included in the *TED brain-blood dataset*. Additionally, 60 healthy controls (HCs) were recruited, with 42 matched controls included in the *HC brain-blood dataset*. Exclusion criteria at each step and reasons for exclusion are detailed.

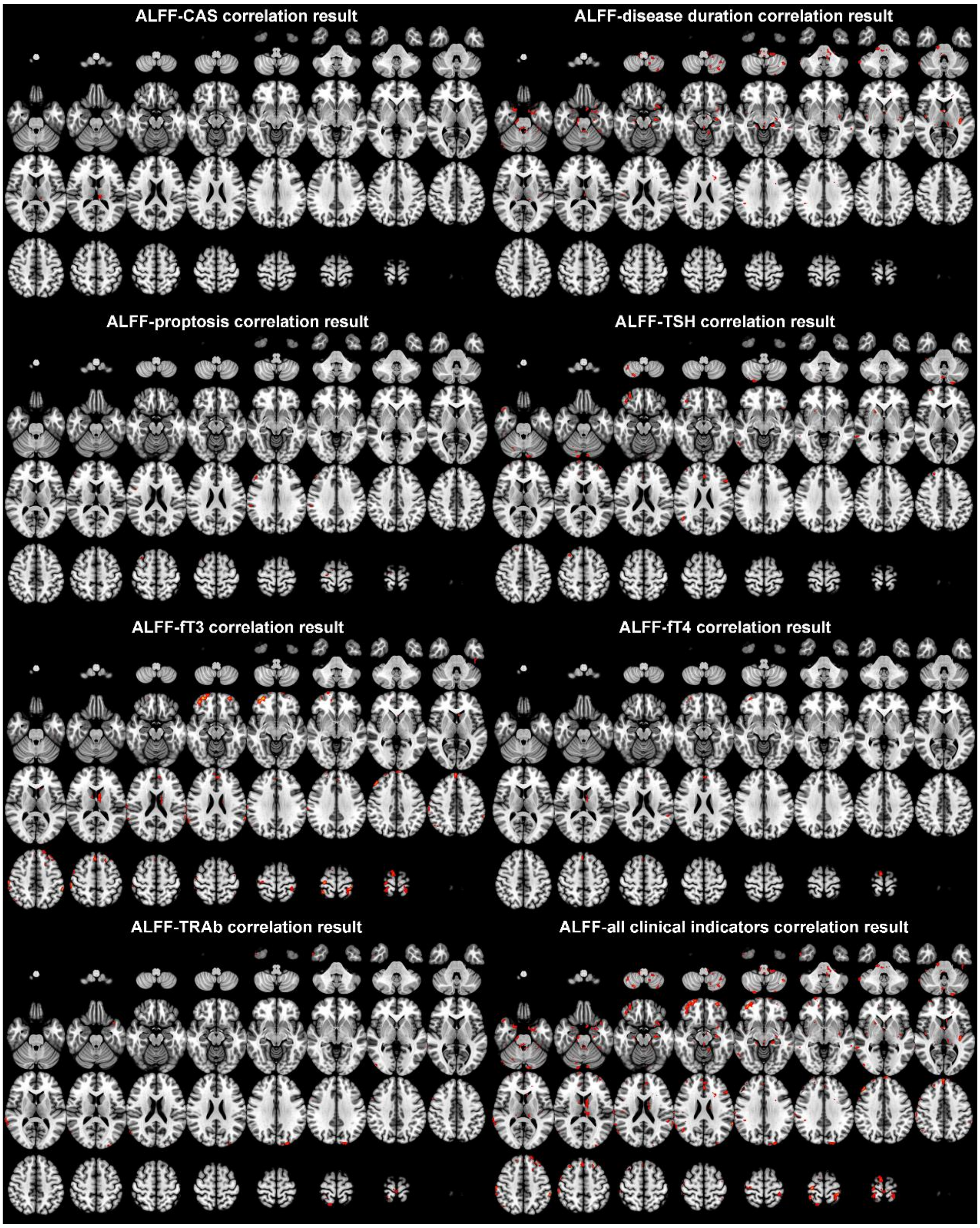

Figure S2. 2D visualization of the significant clusters identified in voxel-wise correlation analyses between ALFF and clinical indicators of *TED brain test dataset*. This figure shows the significant brain regions (highlighted in red) identified in voxel-wise correlation analyses between ALFF and clinical indicators, including CAS, disease duration, proptosis, TSH, fT3, fT4, and TRAb. The clusters shown represent areas where ALFF values significantly correlate with each clinical parameter, with the results thresholded at voxel $p < 0.005$ and cluster-level $p < 0.05$, corrected for GRF multiple comparisons. The brain regions significantly associated with clinical indicators are primarily concentrated in the frontal lobe, subcortex, and brainstem based on the frequency of reported regions (Supplementary Table 1).

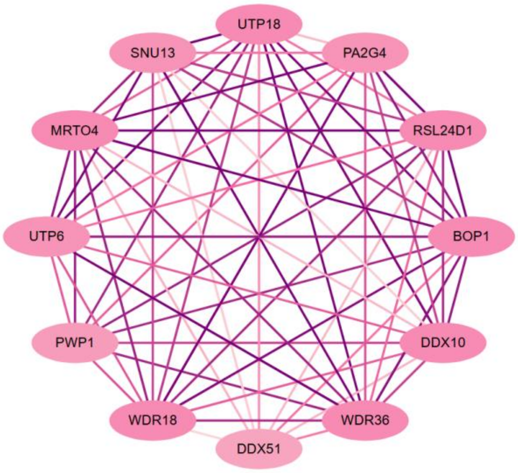

Figure S3. PPI network for MCODE-selected positively ALFF-correlated genes of *TED brain test dataset*. Constructed using the STRING database and analyzed with the MCODE algorithm in Cytoscape. Nodes represent proteins, and edges denote interactions between them. The edge intensity reflects the STRING combined score, indicating the confidence level of each interaction. The genes were primarily enriched in ribosome biogenesis (e.g., UTP18, BOP1) and RNA metabolic regulation (e.g., DDX10, DDX51).

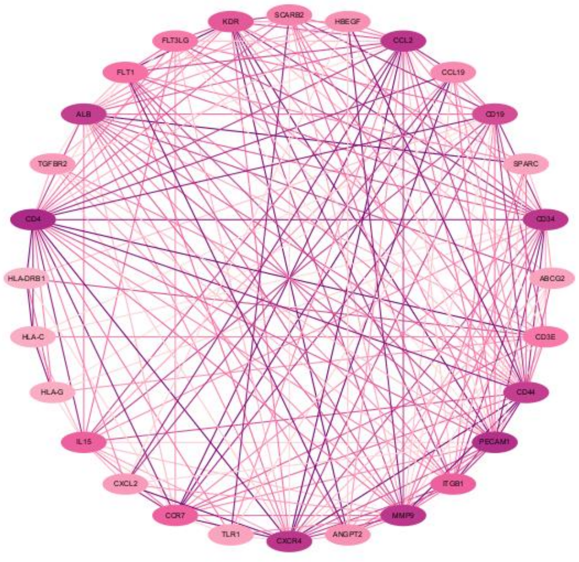

Figure S4. PPI network for MCODE-selected negatively ALFF-correlated genes of *TED brain test dataset*. Constructed using the STRING database and analyzed with the MCODE algorithm in Cytoscape. Nodes represent proteins, and edges denote interactions between them. The edge intensity reflects the STRING combined score, indicating the confidence level of each interaction. The genes were predominantly associated with immune regulation (e.g., CD4 and CD19) and vascular functions (e.g., PECAM1, CD44).

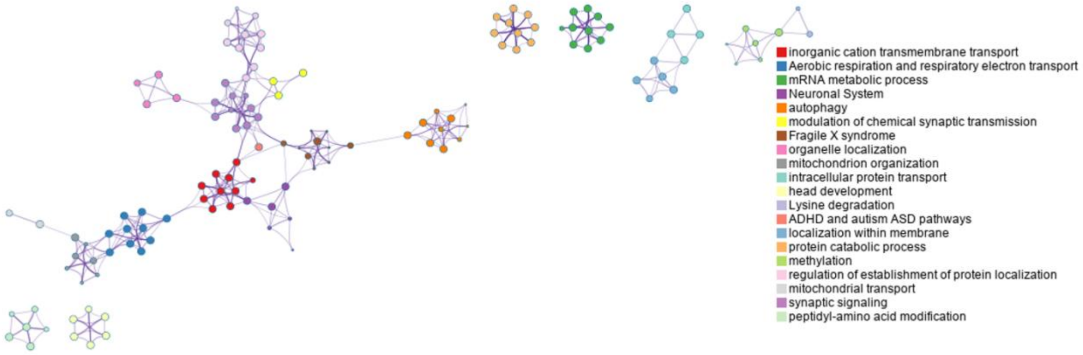

Figure S5. GO term-based pathway enrichment network of positively ALFF-correlated genes of *TED brain test dataset*. The pathway enrichment network of positively correlated genes was constructed using GO terms. Each term is represented by a circle node, where its size is proportional to the number of input genes associated with that term, and its color represents its cluster identity. Terms with a similarity score > 0.3 are linked by an edge (the thickness of the edge represents the similarity score).

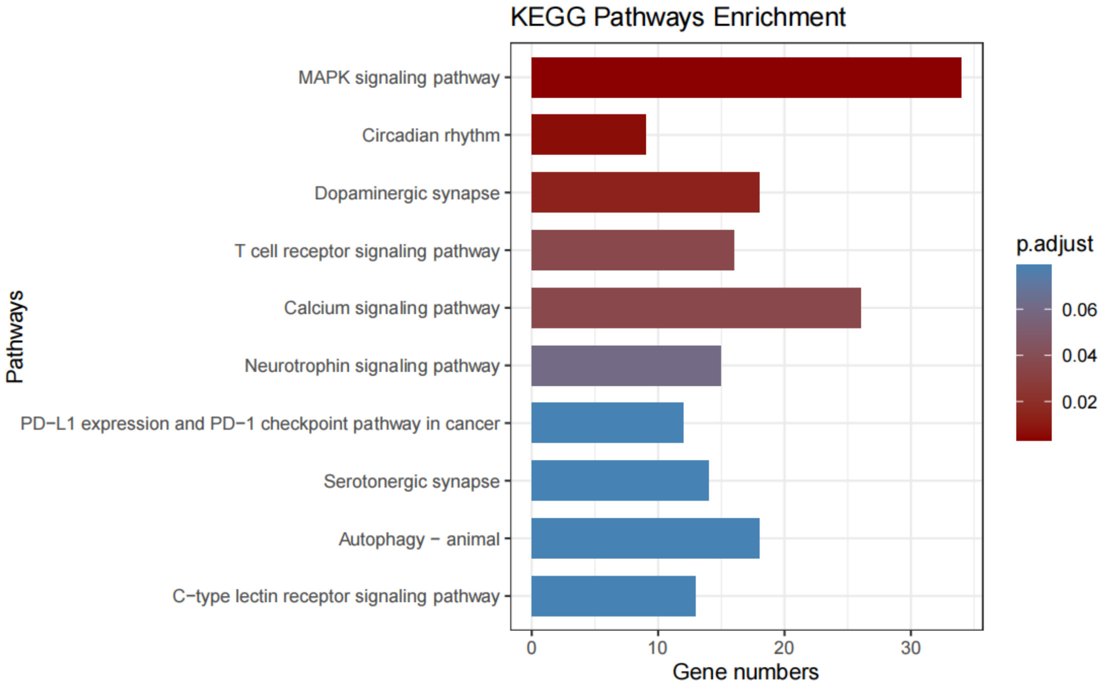

Figure S6. KEGG term-based pathway enrichment analysis of positively ALFF-correlated genes of *TED brain test dataset*. Pathway enrichment analysis of positively correlated genes using KEGG terms. The graph illustrates the top 10 enriched KEGG terms, while color intensity reflects statistical significance (p.adjust). The KEGG enrichment results were mainly on synaptic pathways and calcium signaling pathway.

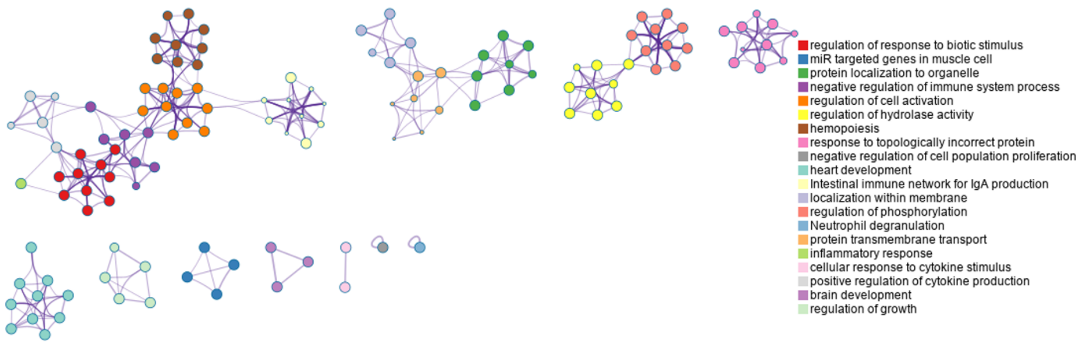

Figure S7. GO term-based pathway enrichment network of negatively ALFF-correlated genes of *TED brain test dataset*. The pathway enrichment network of negatively correlated genes was constructed using GO terms. Each term is represented by a circle node, where its size is proportional to the number of input genes associated with that term, and its color represents its cluster identity. Terms with a similarity score > 0.3 are linked by an edge (the thickness of the edge represents the similarity score).

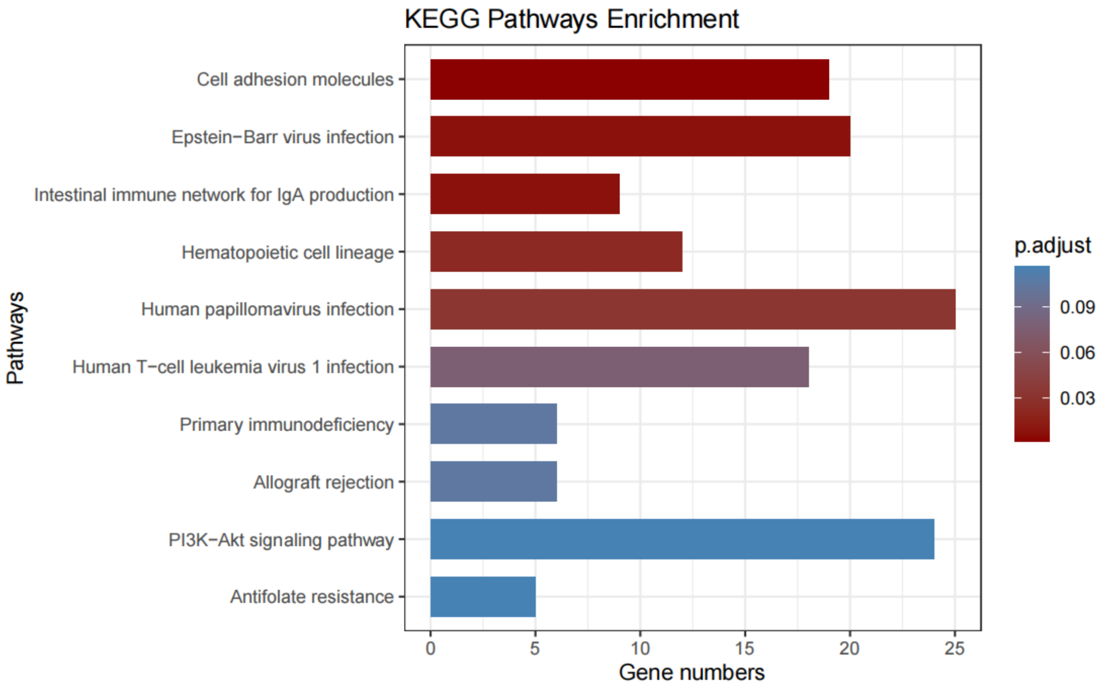

Figure S8. KEGG term-based pathway enrichment analysis of negatively ALFF-correlated genes of *TED brain test dataset*. Pathway enrichment analysis of negatively correlated genes using KEGG terms. The graph illustrates the top 10 enriched KEGG terms, while color intensity reflects statistical significance (p.adjust). The KEGG enrichment results were mainly on immune-related pathways.

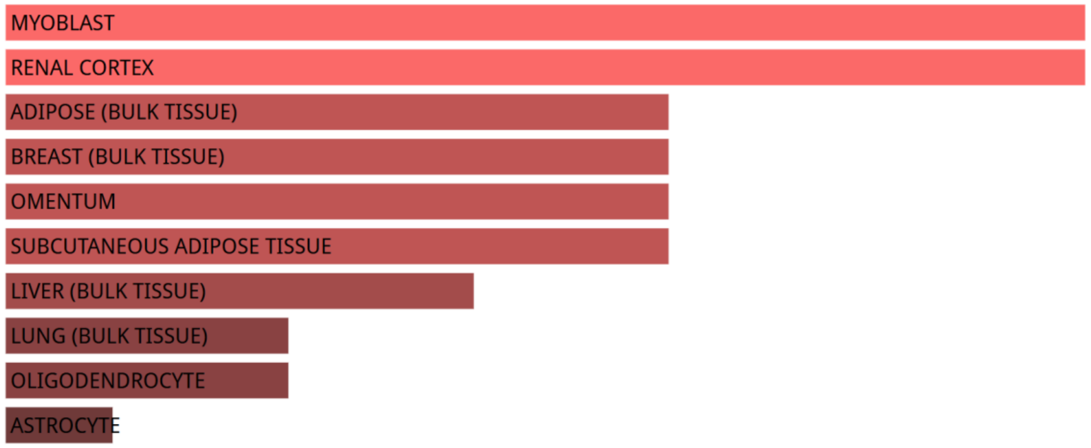

Figure S9. Significant tissue-type enrichment identified using the ARCHS4 dataset. Tissue enrichment analysis of negatively correlated genes using ARCHS4 dataset. The graph illustrates the top 10 enriched tissues, while color intensity reflects statistical significance (p.adjust).

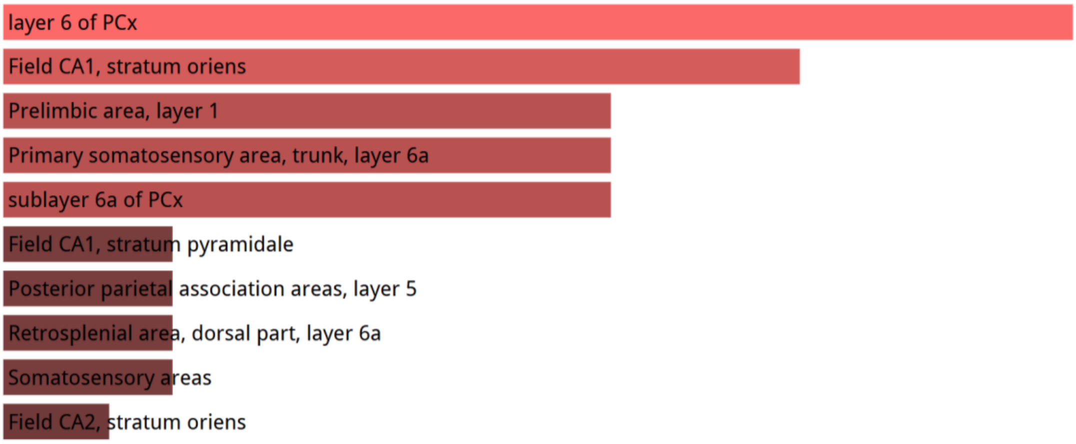

Figure S10. Significant cell-type enrichment identified using the Allen Human Brain Atlas. Cell enrichment analysis of negatively correlated genes using Allen Human Brain Atlas. The graph illustrates the top 10 enriched tissues, while color intensity reflects statistical significance (p.adjust).

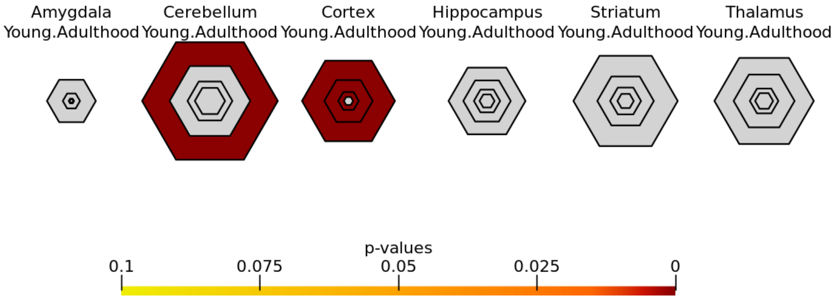

Figure S11. Adult brain region specific expression analyses using the positively ALFF-correlated genes of *TED brain test dataset*. Colored cell types represent significance after multiple testing correction. The sizes of the hexagons denote cell-type specificity across different specificity index probability (pSI) statistic thresholds ranging from 0.05 to 10^{-4}. The outer hexagons correspond to the least specific test for a cell type (pSI threshold = 0.05), whereas the innermost hexagon reflects the most specific test for a cell type (pSI threshold = 10^{-4}). The result showed positively ALFF-correlated genes were mainly expressed in cerebellum and cortex.

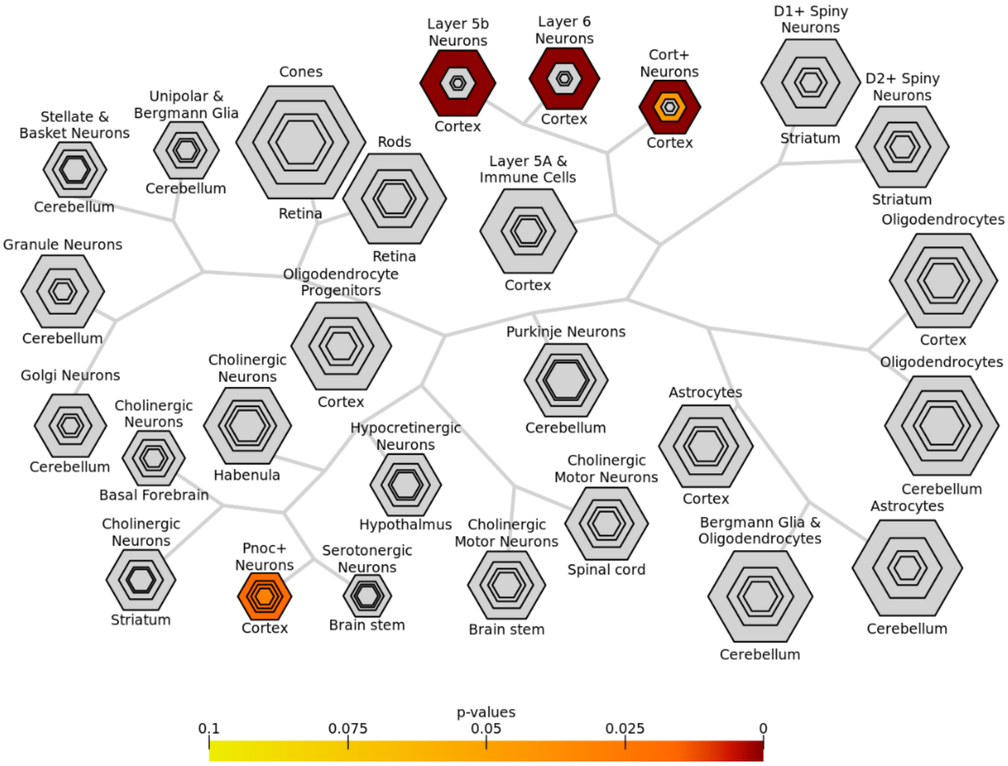

Figure S12. Cell type specific expression analyses using the positively ALFF-correlated genes of *TED brain test dataset*. Colored cell types represent significance after multiple testing correction. The sizes of the hexagons denote cell-type specificity across different pSI statistic thresholds ranging from 0.05 to 10^{-4}. The outer hexagons correspond to the least specific test for a cell type (pSI threshold = 0.05), whereas the innermost hexagon reflects the most specific test for a cell type (pSI threshold = 10^{-4}). The result showed positively ALFF-correlated genes were mainly expressed in cortex neurons.

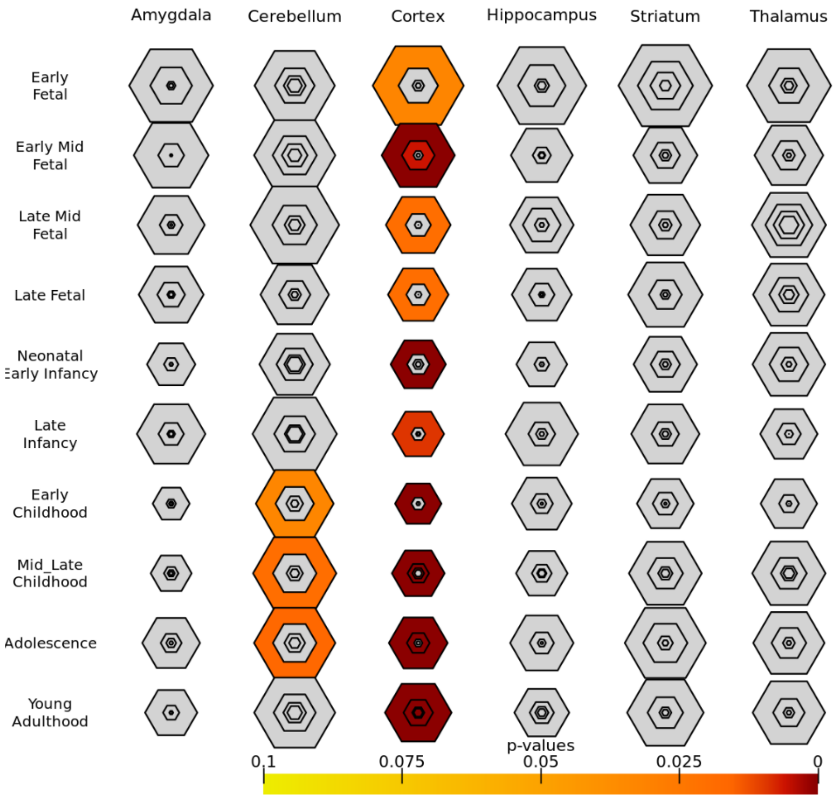

Figure S13. Brain regions specific expression analyses using the positively ALFF-correlated genes across neurodevelopment. Colored cell types represent significance after multiple testing correction. The sizes of the hexagons denote cell-type specificity across different pSI statistic thresholds ranging from 0.05 to 10^{-4}. The outer hexagons correspond to the least specific test for a cell type (pSI threshold = 0.05), whereas the innermost hexagon reflects the most specific test for a cell type (pSI threshold = 10^{-4}). The results showed that positively ALFF-correlated genes were predominantly expressed in the cortex throughout neurodevelopment and in the cerebellum during the later stages of neural development.

.

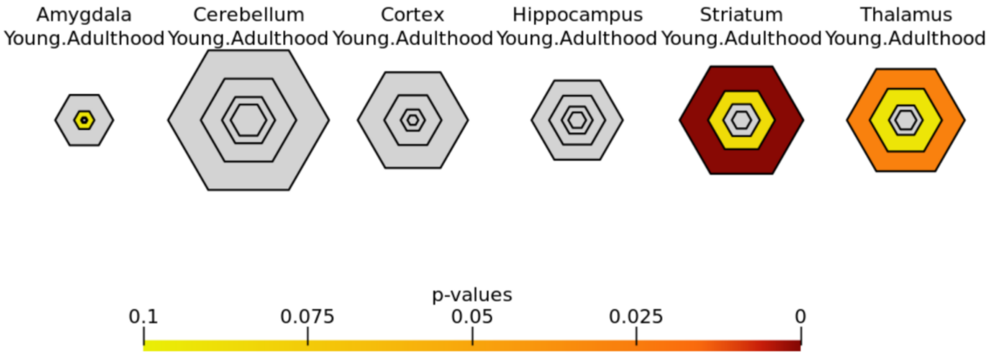

Figure S14. Adult brain region specific expression analyses using the negatively ALFF-correlated genes of *TED brain test dataset*. Colored cell types represent significance after multiple testing correction. The sizes of the hexagons denote cell-type specificity across different specificity index probability (pSI) statistic thresholds ranging from 0.05 to 10^{-4}. The outer hexagons correspond to the least specific test for a cell type (pSI threshold = 0.05), whereas the innermost hexagon reflects the most specific test for a cell type (pSI threshold = 10^{-4}). The result showed negatively ALFF-correlated genes were mainly expressed in striatum and thalamus.

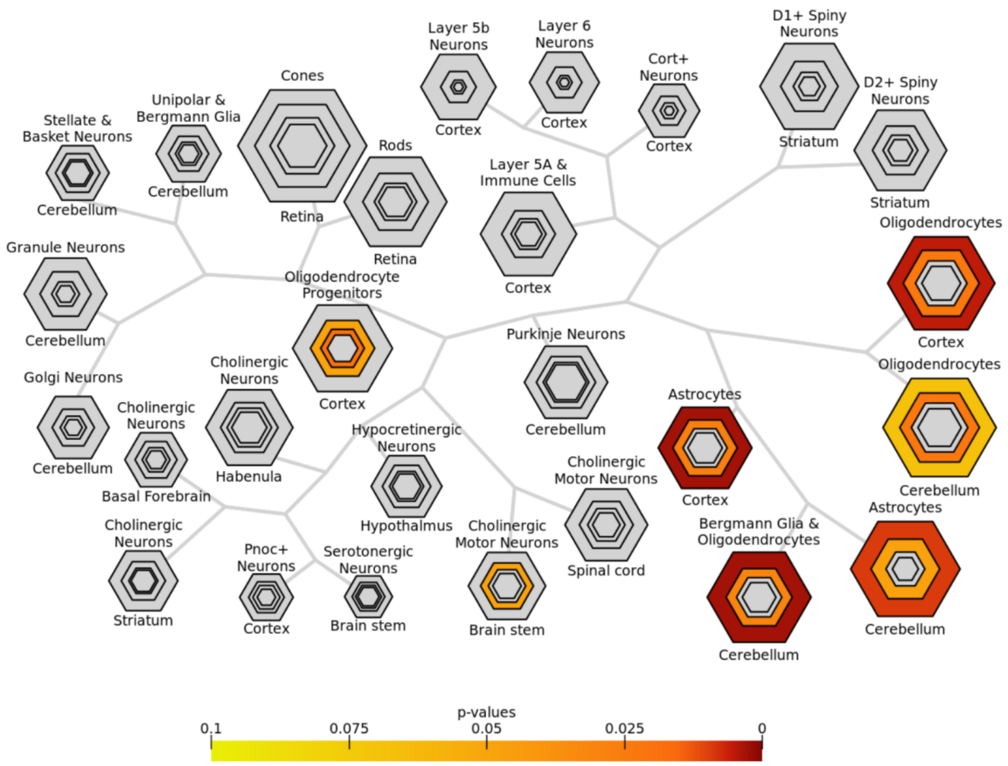

Figure S15. Cell type specific expression analyses using the negatively ALFF-correlated genes of *TED brain test dataset*. Colored cell types represent significance after multiple testing correction. The sizes of the hexagons denote cell-type specificity across different pSI statistic thresholds ranging from 0.05 to 10^{-4}. The outer hexagons correspond to the least specific test for a cell type (pSI threshold = 0.05), whereas the innermost hexagon reflects the most specific test for a cell type (pSI threshold = 10^{-4}). The result showed negatively ALFF-correlated genes were mainly expressed in astrocytes and oligodendrocytes.

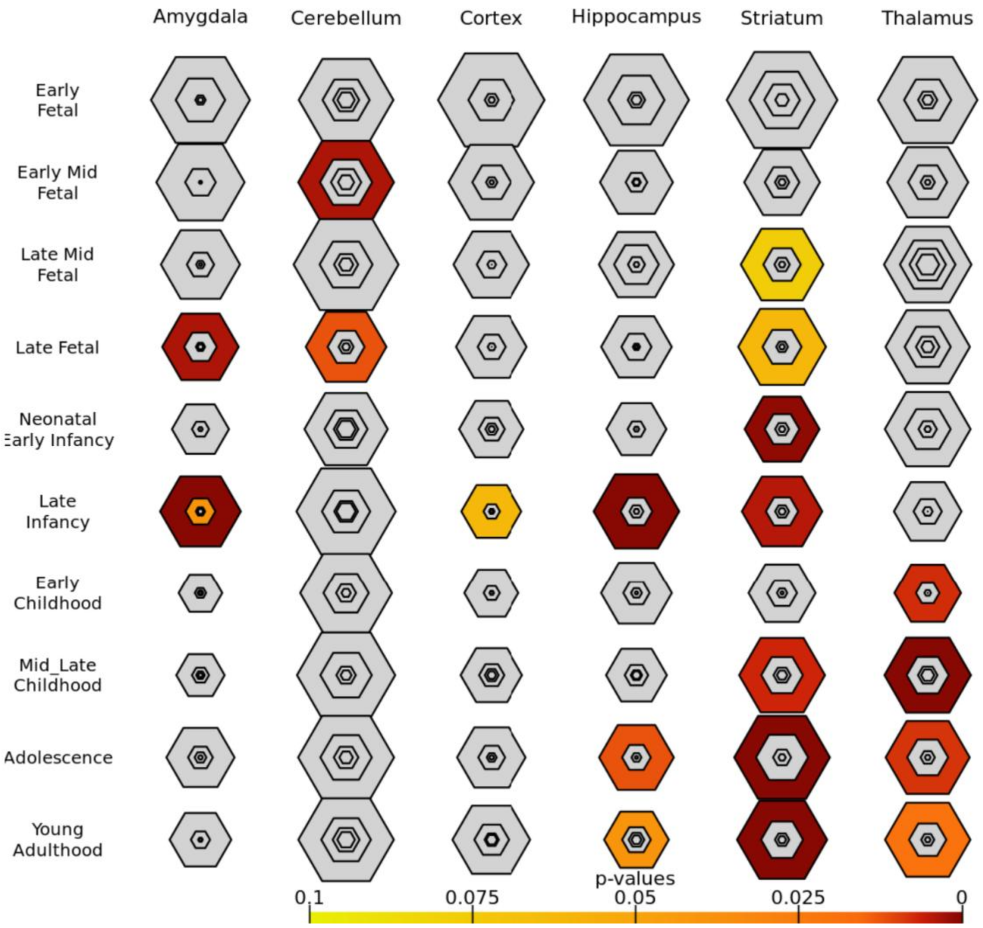

Figure S16. Brain regions specific expression analyses using the negatively ALFF-correlated genes across neurodevelopment. Colored cell types represent significance after multiple testing correction. The sizes of the hexagons denote cell-type specificity across different pSI statistic thresholds ranging from 0.05 to 10^{-4}. The outer hexagons correspond to the least specific test for a cell type (pSI threshold = 0.05), whereas the innermost hexagon reflects the most specific test for a cell type (pSI threshold = 10^{-4}). The results showed that negatively ALFF-correlated genes were mainly expressed in the striatum since late middle fetal and in thalamus during the later stages of neural development.

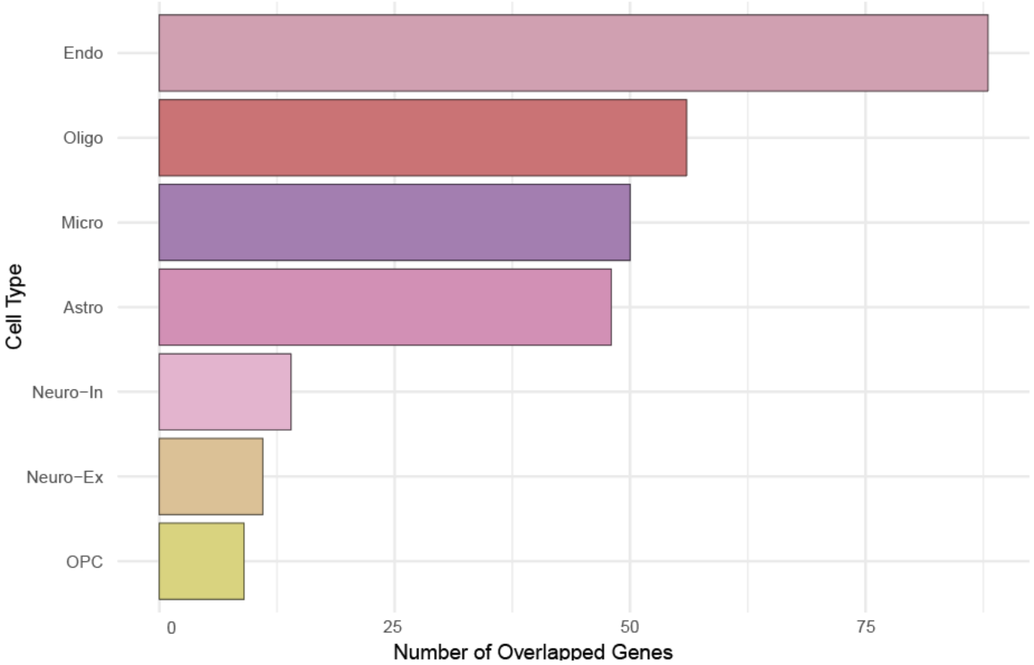

Figure S17. Number of overlapping genes with negatively related genes across seven canonical cell types. The graph illustrates the overlapping genes number across seven canonical cell types. The results showed that negatively ALFF-correlated genes were mainly overlapped in endothelial cells and glial cells.

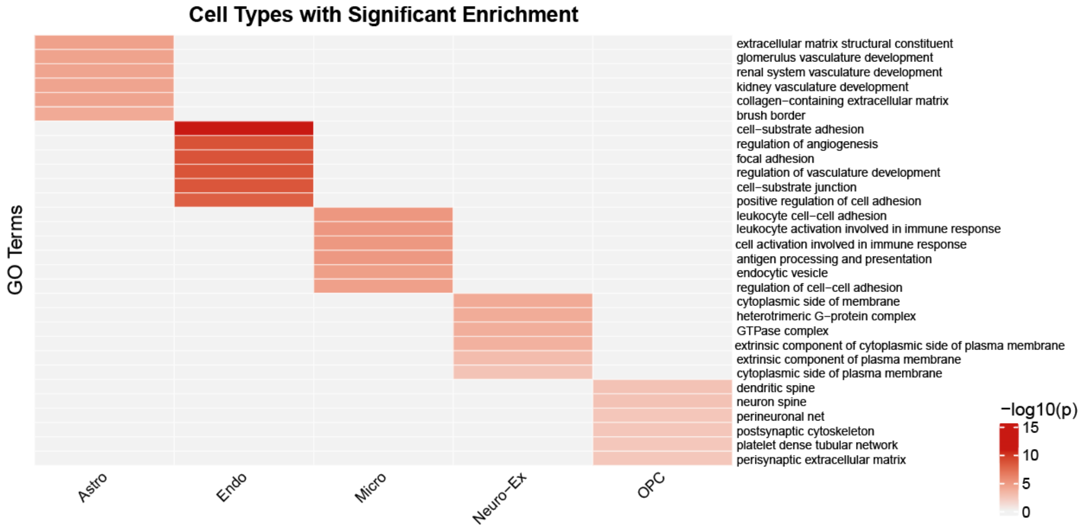

Figure S18. GO term-based pathway enrichment analysis of negatively ALFF-correlated genes across cell types. Pathway enrichment analysis of negatively correlated genes using GO terms. The graph illustrates the top 5 enriched GO terms across cell types, while color intensity reflects statistical significance.

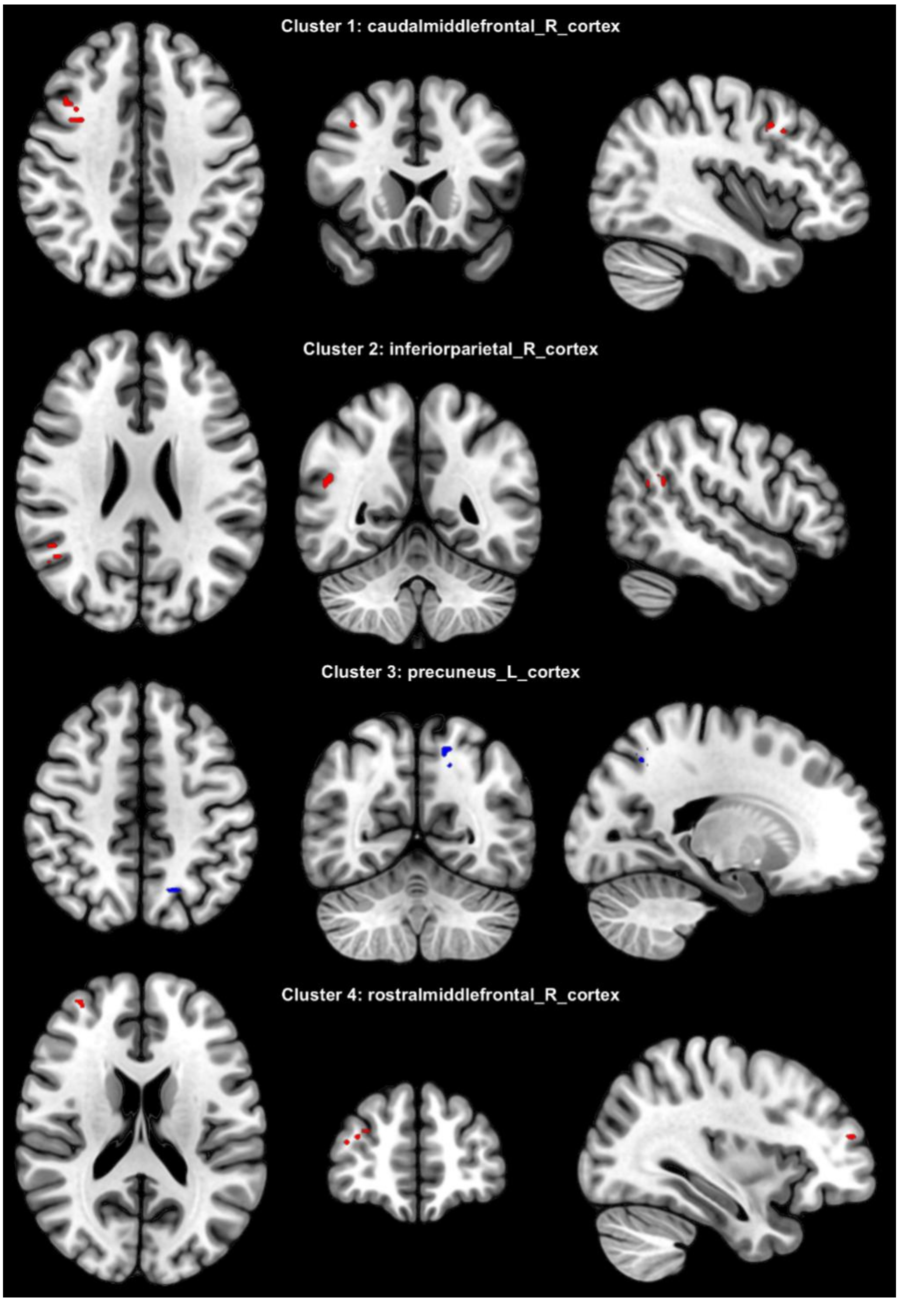

Figure S19. 2D visualization of the four significant clusters identified in whole-brain ALFF analysis of *TED brain-blood dataset*. Each row represents a distinct cluster, overlaid on a standard MNI template in axial, coronal, and sagittal views. The clusters include (1) caudal middle frontal cortex (right), (2) inferior parietal cortex (right), (3) precuneus (left), and (4) rostral middle frontal cortex (right). Red regions indicate increased ALFF, while blue regions indicate decreased ALFF (voxel-level $p < 0.001$, cluster-level $p < 0.05$, GRF corrected). Whole-brain ALFF analysis revealed significant differences in the frontal and parietal lobes between TED patients and HCs, consistent with the TED-related regions previously identified in TED brain test dataset.

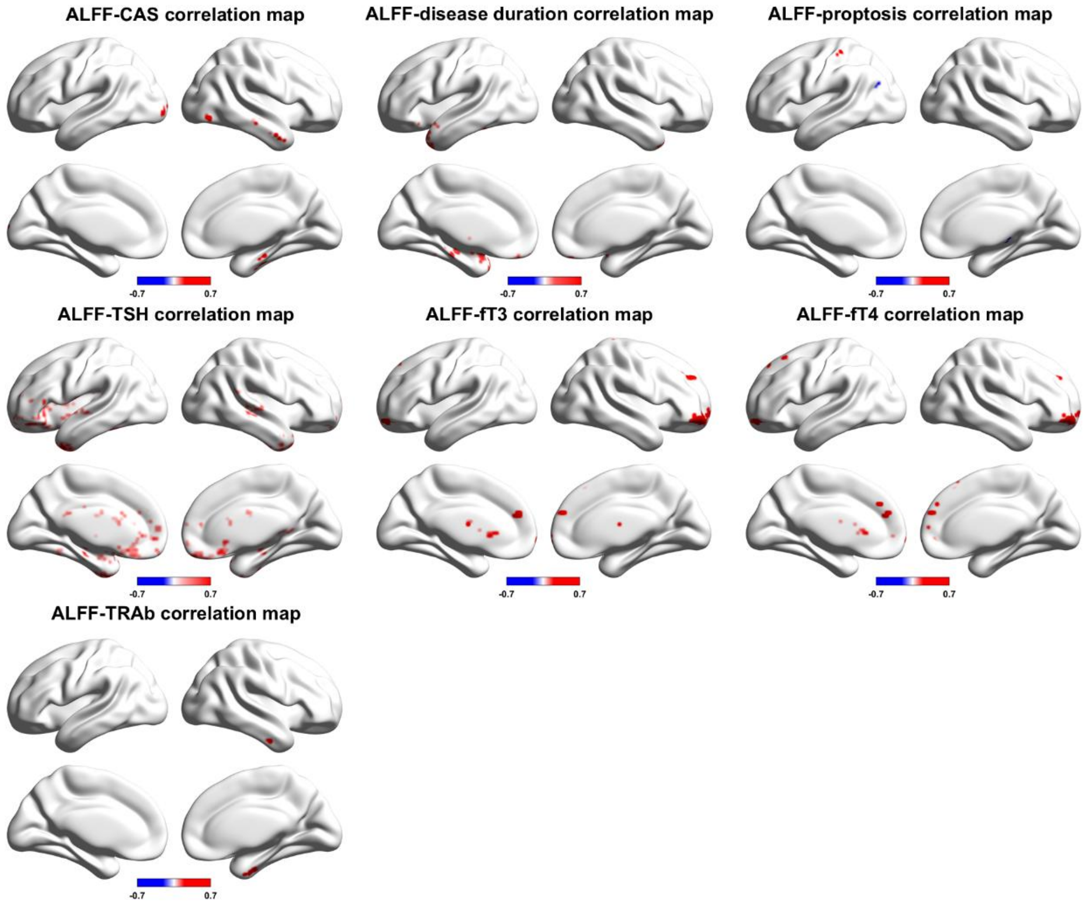

Figure S20. Regional correlation map of ALFF values with clinical indicators in TED brain-blood dataset. The R-map depicted correlations between various clinical indicators and ALFF values in TED patients with PBMC bulk data (voxel-level $p < 0.005$, cluster-level $p < 0.05$, GRF corrected). The color bar represents the correlation coefficient, with red indicating positive correlations and blue indicating negative correlations. The spatial patterns of correlation analyses between ALFF and clinical indicators in the *TED brain-blood dataset* set highly similar to those observed in the *TED brain dataset*, reinforcing the robustness of the disease-associated neural activity alterations.

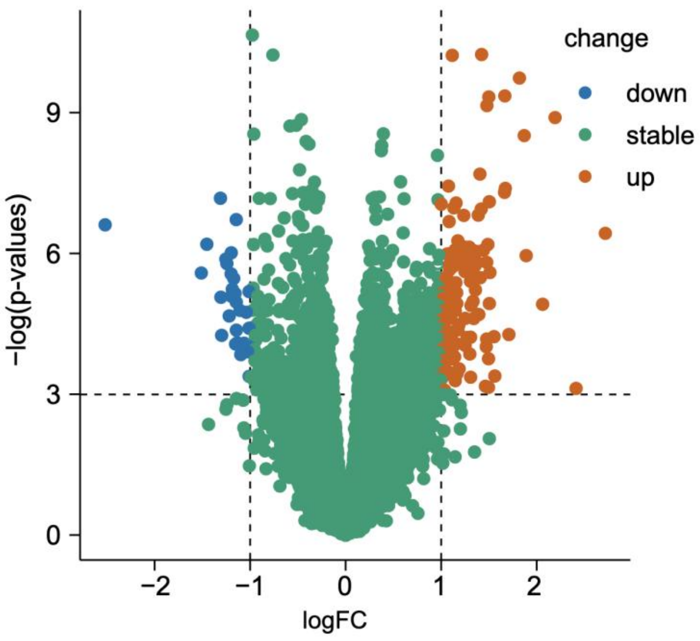

Figure S21. Volcano plot for *PBMC differential expressed genes*. Volcano plot depicting differential gene expression in TED patients versus healthy controls. Significantly dysregulated genes ($p < 0.05$, $|\log2$ fold change$| > 1$) are highlighted. Differential expression analysis between TED patients and HCs identified 65 upregulated and 15 downregulated genes.

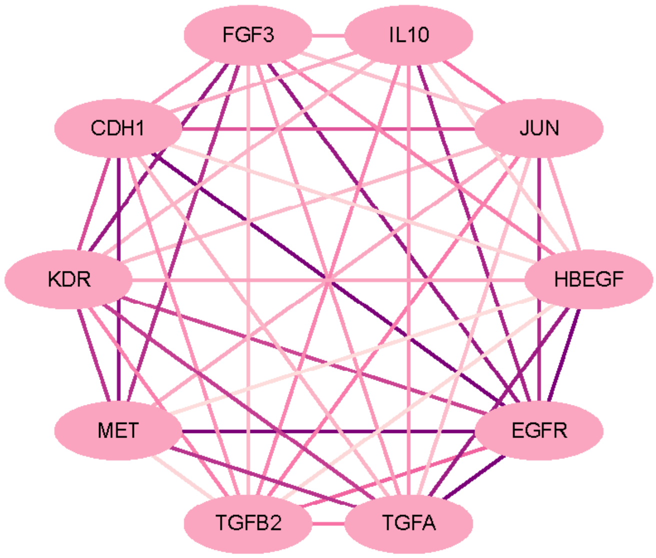

Figure S22. PPI network for MCODE-selected *PBMC differential expressed genes*. Constructed using the STRING database and analyzed with the MCODE algorithm in Cytoscape. Nodes represent proteins, and edges denote interactions between them. The edge intensity reflects the STRING combined score, indicating the confidence level of each interaction. The genes were predominantly associated with cell proliferation (e.g., *EGFR, TGFA*) and immune regulation (e.g., *IL-10, TGFB2*).

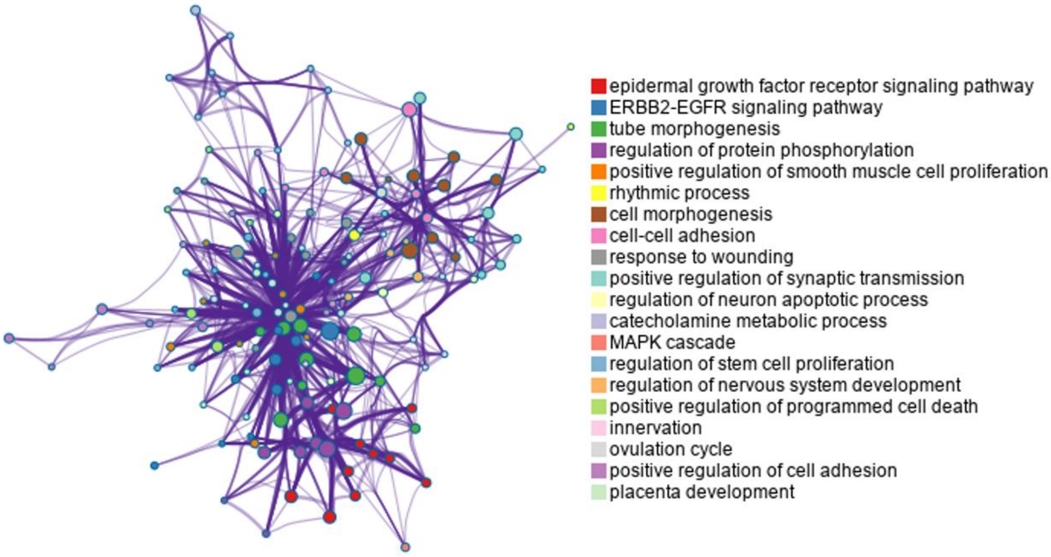

Figure S23. GO term-based pathway enrichment network up-regulated genes in PBMC RNA-seq data. The pathway enrichment network of up-regulated genes was constructed using GO terms. Each term is represented by a circle node, where its size is proportional to the number of input genes associated with that term, and its color represents its cluster identity.. Terms with a similarity score > 0.3 are linked by an edge (the thickness of the edge represents the similarity score).

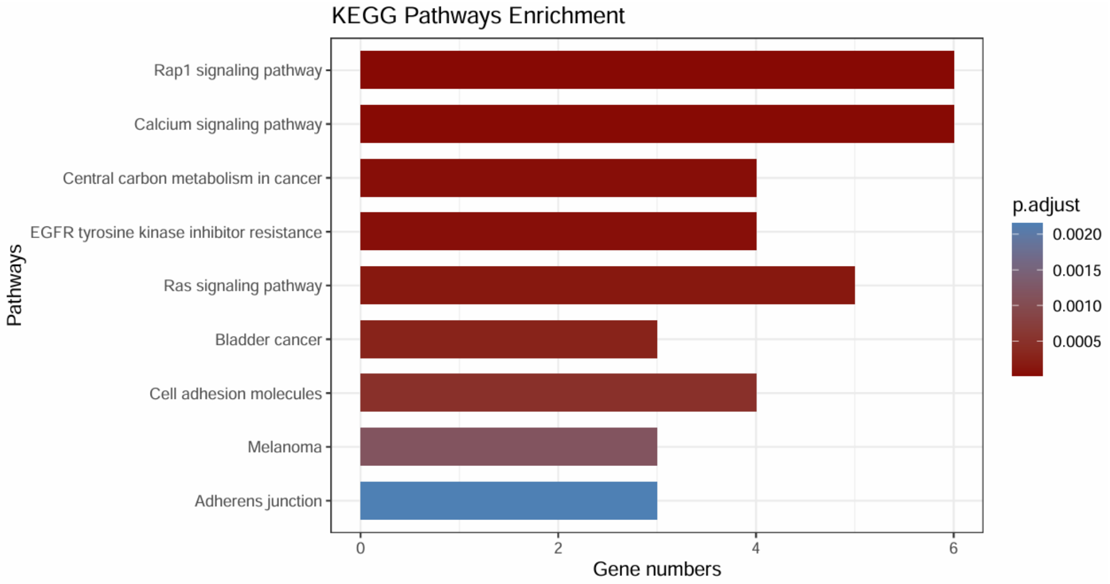

Figure S24. KEGG term-based pathway enrichment analysis of up-regulated genes in PBMC RNA-seq data. Pathway enrichment analysis of negatively correlated genes using KEGG terms. The graph illustrates the top 10 enriched KEGG terms, while color intensity reflects statistical significance (p.adjust). The KEGG enrichment analysis revealed pathways consistent with those identified in the GO analysis.

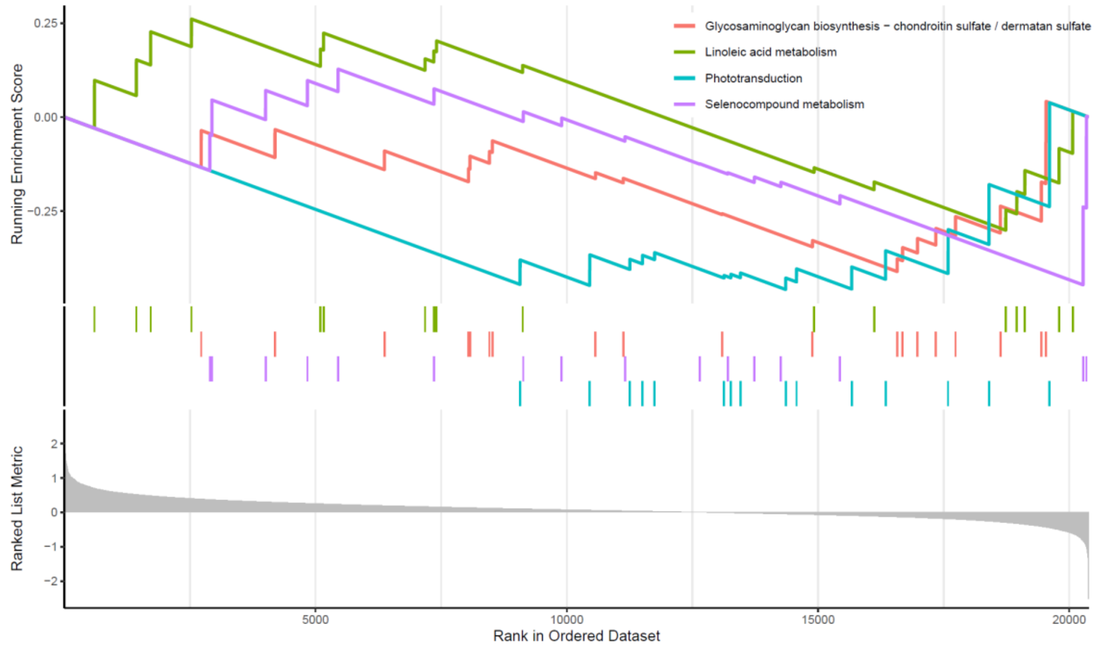

Figure S25. GSVA analysis of down-regulated genes in PBMC RNA-seq data. GSVA of down-regulated pathways in TED patients, highlighting the top four down-regulated enriched pathways. Downregulated pathways were primarily associated with metabolism.

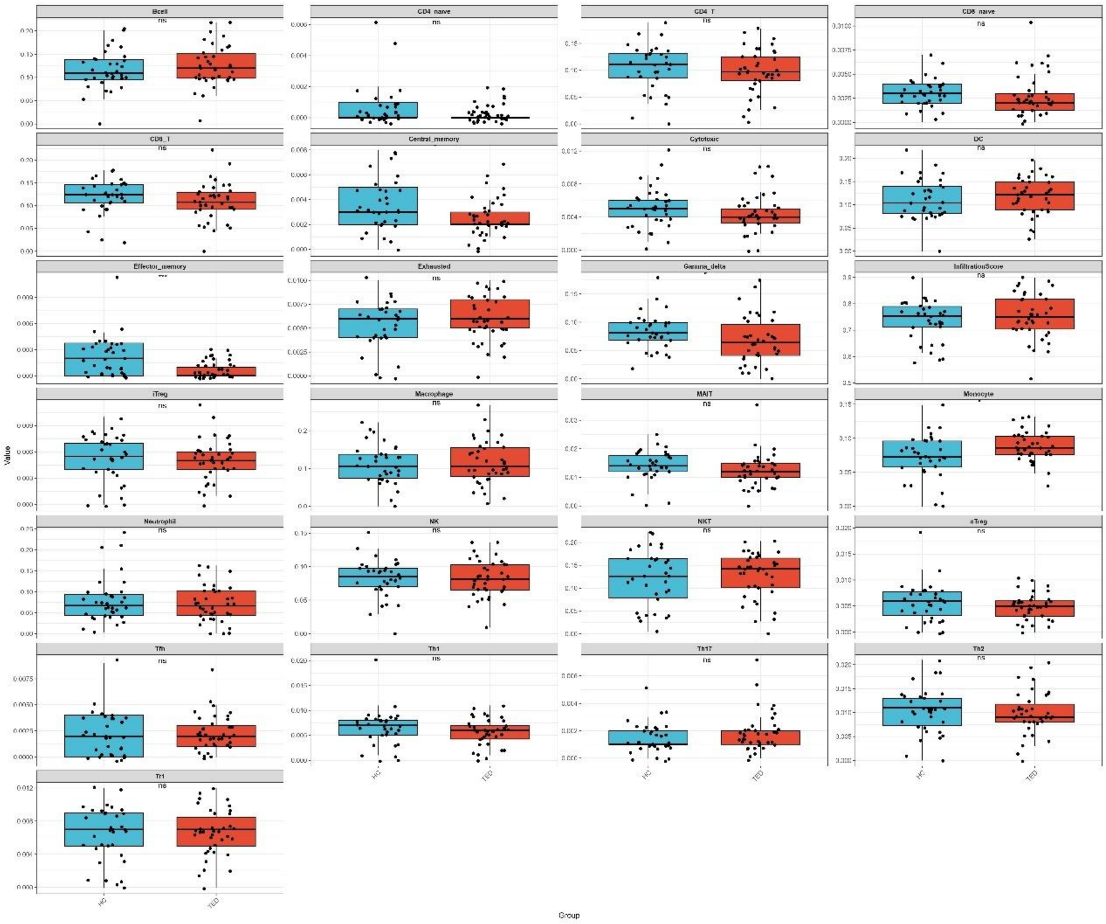

Figure S26. Box plots of immune cell-type infiltration scores for *TED brain-blood dataset* and *HC brain-blood dataset*. Boxplots display the relative proportions of major immune cell populations derived from the immune infiltration analysis. Each box represents the interquartile range (IQR) with a line indicating the median value; whiskers denote 1.5×IQR. Statistical significance between HC and TED groups was assessed using the Wilcoxon rank-sum test. Significant differences in infiltration between *TED brain-blood dataset* and *HC brain-blood dataset* are marked with asterisks (* $p \leq 0.05$, ** $p \leq 0.01$, *** $p \leq 0.001$).

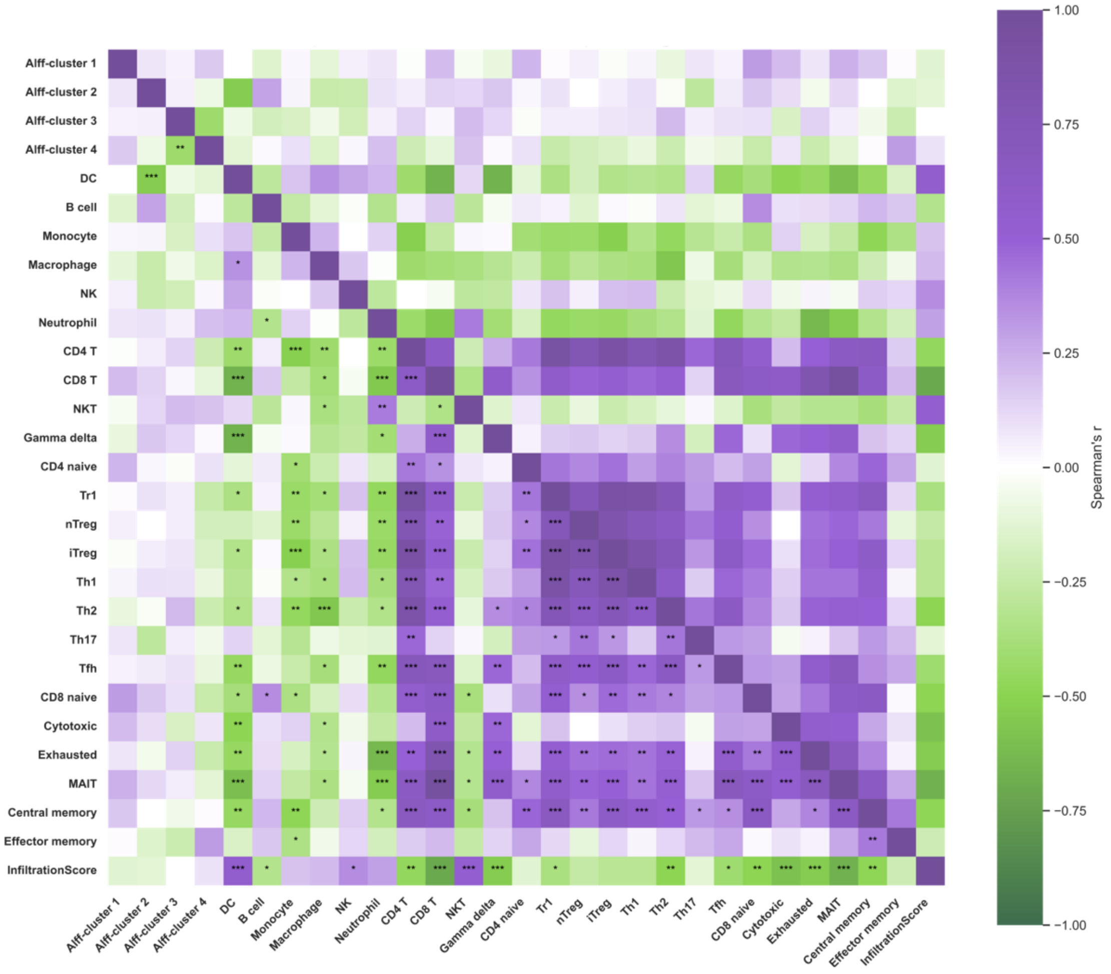

Figure S27. Spearman correlation heatmap of ALFF values from significant brain clusters and immune cell infiltration levels in *TED brain-blood dataset*. Spearman correlation heatmap showing the relationships between ALFF values from the four significant brain clusters from whole brain ALFF analysis and immune cell infiltration levels in *TED brain-blood dataset*. The correlation coefficients are color-coded, with purple indicating positive correlations and green indicating negative correlations. In TED patients, ALFF values in the right inferior parietal cortex were negatively correlated with dendritic cell infiltration. Significant correlations are marked with asterisks (* $p < 0.05$, ** $p < 0.01$, *** $p < 0.001$).

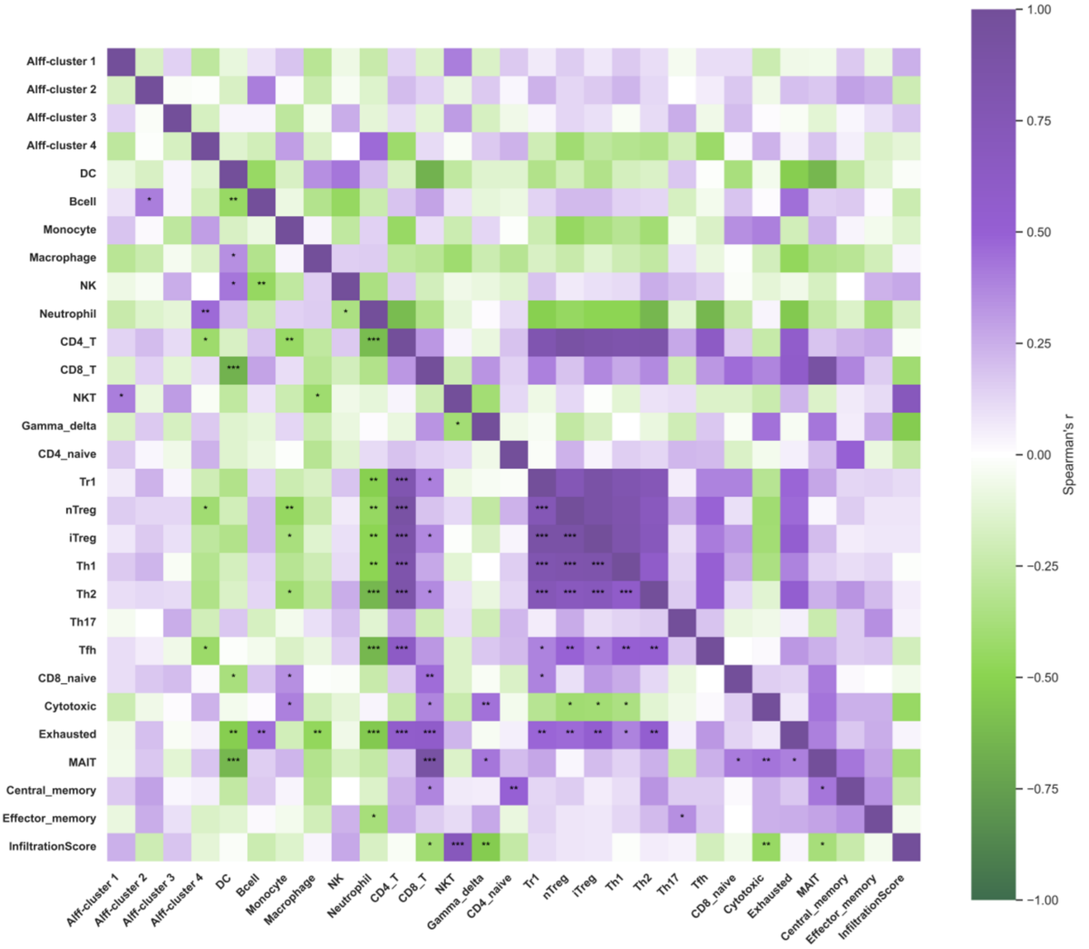

Figure S28. Spearman correlation heatmap of ALFF values from significant brain clusters and immune cell infiltration levels in *HC brain-blood dataset*. Spearman correlation heatmap showing the relationships between ALFF values from the four significant brain clusters from whole brain ALFF analysis and immune cell infiltration levels in HC brain-blood dataset. The correlation coefficients are color-coded, with purple indicating positive correlations and green indicating negative correlations. In HCs, ALFF in the right caudal middle frontal cortex was positively associated with NKT cells, and ALFF in the right inferior parietal cortex was positively correlated with B cells. Significant correlations are marked with asterisks (* $p < 0.05$, ** $p < 0.01$, *** $p < 0.001$).

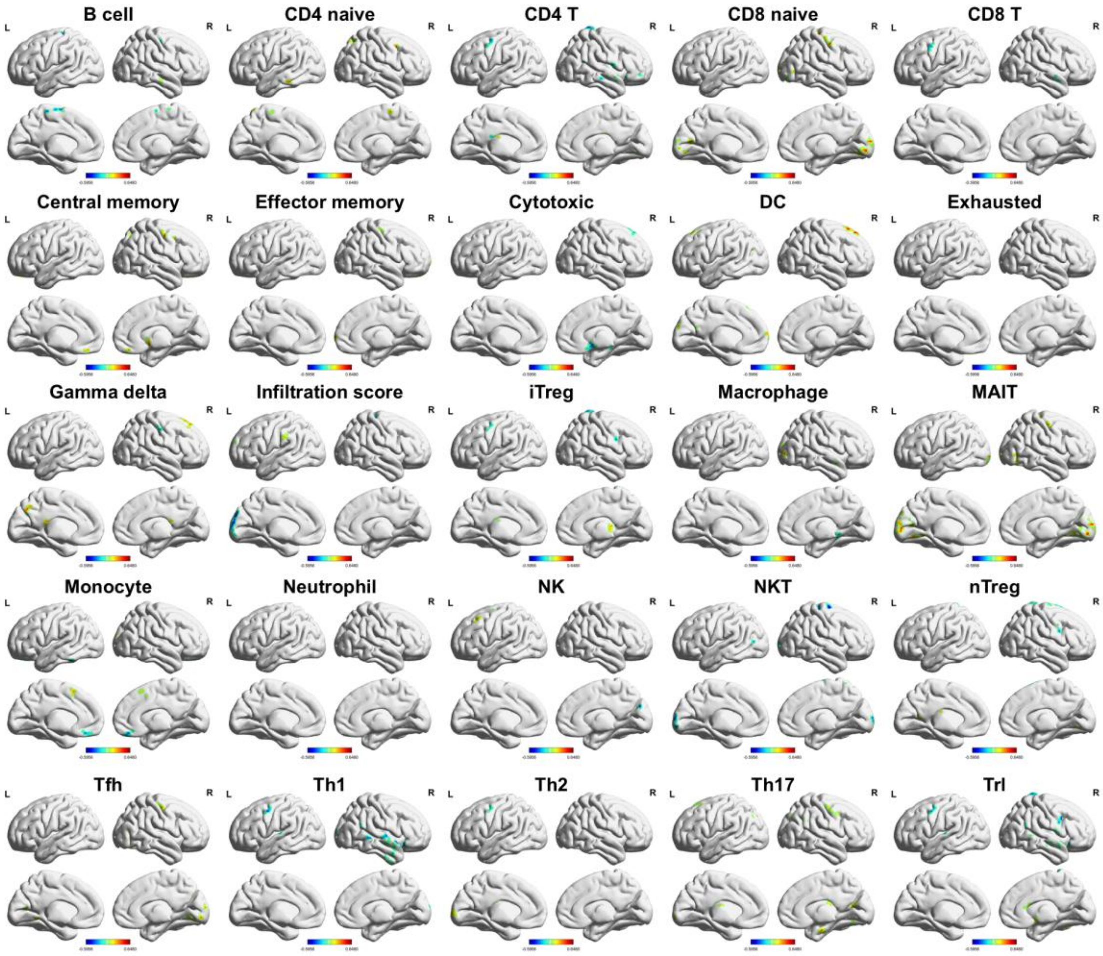

Figure S29. Voxel-wise correlation maps of whole-brain ALFF values with immune cell infiltration levels in TED brain-blood dataset. Voxel-wise correlation maps showing significant associations between whole-brain ALFF values and immune cell infiltration levels in *TED brain-blood dataset*. Each panel represents a specific immune cell type, with significant correlation coefficients (r) visualized (voxel-level $p < 0.005$, cluster-level $p < 0.05$, GRF corrected). Warm colors indicate positive correlations, while cool colors indicate negative correlations. For T cell subset infiltration levels, they were significantly correlated with ALFF values in widespread cortical and subcortical regions, predominantly localized in the frontal area, followed by subcortical, parietal, temporal areas, brainstem, occipital area and insular cortices in TED.

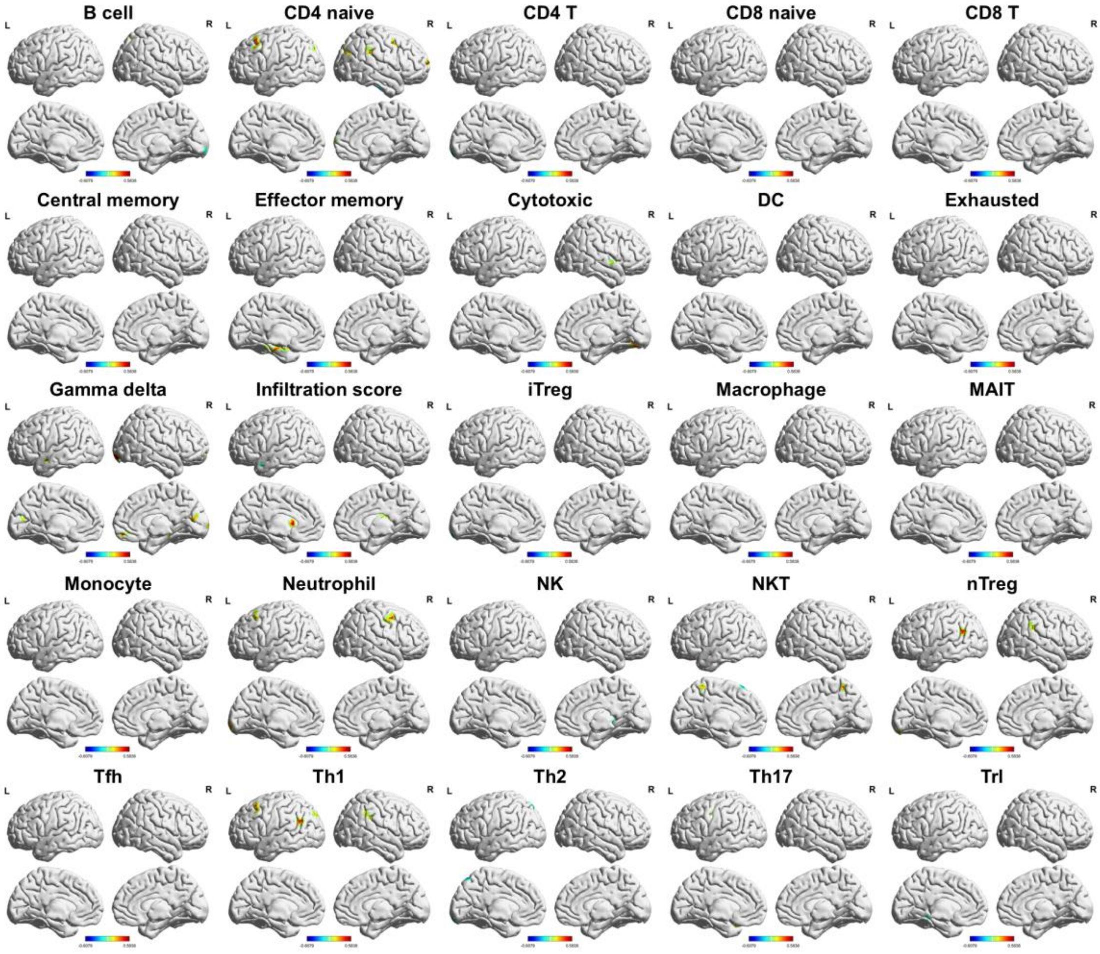

Figure S30. Voxel-wise correlation maps of whole-brain ALFF values with immune cell infiltration levels in *HC brain-blood dataset*. Voxel-wise correlation maps showing significant associations between whole-brain ALFF values and immune cell infiltration levels in HC brain-blood dataset. Each panel represents a specific immune cell type, with significant correlation coefficients (r) visualized (voxel-level p < 0.005, cluster-level p < 0.05, GRF corrected). Warm colors indicate positive correlations, while cool colors indicate negative correlations. HCs displayed different patterns of spatial correlation between whole brain ALFF and immune cell infiltration levels compared to TED, suggesting that the observed brain-immune correlation patterns in TED are disease-specific.

Supplementary Tables S1-S5:

Table S1. Significant correlation between whole-brain ALFF and clinical index in *TED brain test dataset* (voxel $p < 0.005$, cluster $p < 0.05$, cluster-level GRF corrected)

Condition	Brain regions of peak voxel (DK atlas)	R value	Cluster size	MNI coordinates of peak voxel		
				X	Y	Z
CAS	thalamusproper_L_subcortex_brainstem	0.367003	15	-12	-21	15
	inferiortemporal_L_cortex	-0.365955	12	-54	-54	-26
	superiorfrontal_L_cortex	-0.292299	7	-5	-12	58
	supramarginal_L_cortex	-0.363766	6	-42	-48	36
Disease duration	brainstem_B_subcortex_brainstem	0.454024	49	-9	-45	-42
	amygdala_L_subcortex_brainstem	0.463846	42	-18	-3	-27
	brainstem_B_subcortex_brainstem	0.444819	33	12	-24	-36
	brainstem_B_subcortex_brainstem	0.382439	33	31	29	18
	insula_L_cortex	0.473474	28	-37	-20	6
	hippocampus_L_subcortex_brainstem	0.451977	22	-24	-18	-24
	entorhinal_R_cortex	0.440327	17	18	3	-27
	brainstem_B_subcortex_brainstem	0.412476	15	9	-39	-45
	thalamusproper_L_subcortex_brainstem	0.395278	14	-7	-21	15
	fusiform_L_cortex	0.33272	13	-30	-42	-21
	hippocampus_L_subcortex_brainstem	0.408902	13	-21	-30	-9
	hippocampus_R_subcortex_brainstem	0.356388	12	24	-27	-12
	brainstem_B_subcortex_brainstem	0.408568	11	0	-33	-6
	supramarginal_R_cortex	0.39097	11	46	-39	27
	brainstem_B_subcortex_brainstem	0.38067	10	6	-42	-60
	inferiortemporal_L_cortex	0.354079	9	-32	6	-45
	insula_L_cortex	0.392654	9	-33	12	-18
	thalamusproper_R_subcortex_brainstem	0.372556	9	18	-27	9
	insula_R_cortex	0.360214	8	39	-6	-12
	insula_R_cortex	0.461977	8	42	-12	6
	hippocampus_R_subcortex_brainstem	0.371899	7	21	-18	-21
	middletemporal_L_cortex	0.427427	7	-60	-36	-9
	brainstem_B_subcortex_brainstem	0.36294	6	-15	-27	-21
Proptosis	superiortemporal_L_cortex	-0.348603	14	-36	6	-26
	rostralmiddlefrontal_R_cortex	0.361731	10	45	36	30
	supramarginal_R_cortex	0.338099	9	57	-33	33

	caudalmiddlefrontal_R_cortex	0.366401	8	33	18	60
	precentral_R_cortex	0.337687	8	21	-21	72
	rostralmiddlefrontal_R_cortex	0.308721	6	48	42	15
	parsopercularis_R_cortex	0.374995	6	48	6	18
TSH	parsorbitalis_R_cortex	0.465601	10	39	39	-15
	parsorbitalis_R_cortex	0.349269	10	36	48	-15
	rostralanteriorcingulate_R_cortex	0.492627	10	3	39	18
	superiorfrontal_L_cortex	0.460771	10	-3	44	27
	rostralmiddlefrontal_L_cortex	0.395508	8	-39	54	15
	inferiorparietal_R_cortex	0.427386	8	48	-63	24
	fusiform_L_cortex	-0.410149	7	-30	-12	-42
	superiortemporal_R_cortex	0.487397	7	48	18	-27
	putamen_R_subcortex_brainstem	0.368399	7	21	15	0
	bankssts_R_cortex	0.338348	7	60	-42	0
	superiorfrontal_R_cortex	0.449694	7	6	69	12
	rostralmiddlefrontal_L_cortex	0.370087	7	-52	24	27
	caudalmiddlefrontal_R_cortex	0.398385	7	33	24	54
	parsorbitalis_L_cortex	0.38405	6	-45	21	-12
	inferiortemporal_R_cortex	0.438189	6	58	-63	-9
	rostralmiddlefrontal_R_cortex	0.37091	6	27	66	9
	rostralmiddlefrontal_R_cortex	0.342029	6	39	45	30
	superiorfrontal_R_cortex	0.381071	6	21	42	45
fT3	rostralmiddlefrontal_R_cortex	0.756953	75	30	57	-12
	precentral_R_cortex	0.499768	66	24	-24	74
	postcentral_L_cortex	0.479432	52	-33	-36	72
	superiorfrontal_R_cortex	0.699697	49	-9	59	39
	caudate_L_subcortex_brainstem	0.468551	38	-9	4	13
	supramarginal_R_cortex	0.580822	35	60	-28	51
	postcentral_R_cortex	0.544431	20	68	-18	23
	superiorfrontal_R_cortex	0.584235	20	6	-3	75
	caudalmiddlefrontal_L_cortex	0.40731	18	-36	27	51
	rostralmiddlefrontal_R_cortex	0.461657	16	20	66	-6
	rostralmiddlefrontal_L_cortex	0.595917	15	-30	57	-12
	fusiform_L_cortex	0.45348	13	-42	-24	-29
	rostralmiddlefrontal_R_cortex	0.471588	13	21	55	36
	supramarginal_R_cortex	0.452828	12	66	-48	18
	rostralmiddlefrontal_R_cortex	0.557707	11	45	36	36

	superiorfrontal_L_cortex	0.483386	10	-6	45	21
	superiorfrontal_L_cortex	0.484491	10	-21	27	54
	supramarginal_R_cortex	0.43469	9	66	-21	30
	precentral_L_cortex	0.437827	8	-9	-27	78
	rostralmiddlefrontal_L_cortex	0.426525	7	-15	69	-9
	caudate_R_subcortex_brainstem	0.438306	7	9	6	15
	superiortemporal_L_cortex	0.589667	7	-66	-45	21
	superiorfrontal_L_cortex	0.366633	7	3	40	30
	precentral_L_cortex	0.464991	7	-42	-9	63
	medialorbitofrontal_R_cortex	0.370218	6	12	52	-3
	superiorfrontal_R_cortex	0.348322	6	3	57	27
	inferiorparietal_R_cortex	0.393746	6	51	-63	45
	supramarginal_L_cortex	0.590825	6	60	-38	48
fT4	rostralmiddlefrontal_R_cortex	0.514417	20	30	57	-12
	superiorfrontal_R_cortex	0.480789	14	6	-3	75
	thalamusproper_L_subcortex_brainstem	0.339599	12	-9	-6	15
	superiortemporal_L_cortex	0.355358	7	-57	-39	21
	superiorfrontal_L_cortex	0.298615	7	-6	45	24
	superiorfrontal_R_cortex	0.407848	6	3	30	54
TRAb	superiorparietal_L_cortex	0.408888	32	-22	-90	36
	precentral_R_cortex	0.317113	16	5	-30	78
	superiortemporal_R_cortex	0.406781	15	69	-42	9
	middletemporal_R_cortex	0.338001	11	51	6	-42
	lateraloccipital_L_cortex	0.350143	9	-27	-96	15
	inferiorparietal_R_cortex	0.324111	8	57	-69	21
	superiorparietal_R_cortex	0.32139	8	15	-51	75
	superiortemporal_L_cortex	0.384174	7	-42	12	-21
	lateraloccipital_R_cortex	0.276433	7	45	-84	0
	superiorparietal_R_cortex	0.321757	7	38	-57	60

ALFF: amplitude of low-frequency fluctuation; TED: thyroid eye disease; DK: Desikan–Killiany; MNI: Montreal Neurological Institute; CAS: clinical activity score; TSH: thyroid-stimulating hormone; fT3: free triiodothyronine; fT4: free thyroxine; TRAb: thyroid-stimulating hormone receptor antibodies.

Table S2. Brain regions selected based on whole-brain ALFF and clinical index correlation analysis in the *TED brain test dataset*

ID	DK-label
21	postcentral_L_cortex
26	rostralmiddlefrontal_L_cortex
27	superiorfrontal_L_cortex
28	superiorparietal_L_cortex
33	transversetemporal_L_cortex
34	insula_L_cortex
35	thalamusproper_L_subcortex_brainstem
36	caudate_L_subcortex_brainstem
37	putamen_L_subcortex_brainstem
40	hippocampus_L_subcortex_brainstem
59	parsorbitalis_R_cortex
62	postcentral_R_cortex
64	precentral_R_cortex
67	rostralmiddlefrontal_R_cortex
68	superiorfrontal_R_cortex
69	superiorparietal_R_cortex
71	supramarginal_R_cortex
83	brainstem_B_subcortex_brainstem

ALFF: amplitude of low-frequency fluctuation; TED: thyroid eye disease; DK: Desikan–Killiany.

Table S3. ssGSEA mapping of selective pathway of whole brain regions in the *TED brain test dataset*

	Regulation of leukocyte cell-cell adhesion	Positive regulation of vasculogenesis	Modulation of chemical synaptic transmission	Regulation of T cell activation	Regulation of trans-synaptic signaling	Regulation of vasculature development	Regulation of vasculogenesis	Response to light stimulus	Vesicle-mediated transport in synapse
accumbensarea_L	0.05511	0.04091	-0.30827	0.1185	-0.31146	0.56055	0.23384	-0.09944	-0.35992
accumbensarea_R	0.16254	-0.21263	-0.18028	0.07373	-0.24042	0.68486	-0.11404	0.02481	-0.42235
amygdala_L	0.12565	0.11931	-0.26118	0.16362	0.00821	0.69138	0.23884	-0.02518	0.0903
amygdala_R	-0.05216	-0.02223	-0.02157	-0.05369	0.14245	0.793	0.11504	0.02877	0.23836
bankssts_L	-0.17908	0.42146	0.27317	-0.17308	0.15382	-0.64637	0.28733	-0.09104	0.2991
bankssts_R	-0.07804	-0.31973	0.24111	-0.11557	0.11559	-0.55761	-0.41974	0.06562	0.10076
brainstem_B	0.10514	0.45759	-0.25177	0.0963	-0.2804	0.13995	0.46497	-0.21275	-0.22164
caudalanteriorcingulate_L	0.10858	0.20584	-0.41051	0.0548	-0.08202	0.75446	0.26828	-0.10463	-0.19772
caudalanteriorcingulate_R	0.0917	-0.1464	-0.02307	0.02317	0.04556	0.38353	-0.1078	0.05029	-0.07141
caudalmiddlefrontal_L	-0.03391	0.51401	0.12528	-0.03564	0.08304	0.41608	0.4881	-0.09186	0.14785
caudalmiddlefrontal_R	-0.123	-0.39539	-0.02524	-0.06146	0.17493	-0.58037	-0.45311	-0.03753	0.16379
caudate_L	0.18229	0.40072	-0.20627	0.19592	-0.19177	0.72497	0.53894	-0.01712	-0.43049
caudate_R	0.12566	0.35385	-0.11984	0.06446	-0.08179	0.69983	0.46863	0.03143	-0.39033
cuneus_L	-0.14115	0.09372	0.11995	-0.16894	-0.09384	-0.80622	0.05506	0.12938	0.02557
cuneus_R	-0.11855	-0.49033	0.32561	-0.10676	0.19603	-0.89569	-0.45616	0.12099	0.29761
entorhinal_L	0.04097	0.0502	-0.06405	0.07649	0.12818	0.79144	0.13199	-0.11655	0.18095
entorhinal_R	-0.06119	-0.25	0.02334	0.00537	0.2004	0.65984	-0.01165	-0.09674	0.22532
frontalpole_L	0.22583	0.40171	-0.40619	0.14866	-0.25938	0.5271	0.20263	-0.0538	-0.27371
frontalpole_R	-0.04203	-0.29919	0.13171	0.04324	0.22975	0.25274	-0.26786	-0.00587	0.2073
fusiform_L	-0.11733	0.09713	0.3155	-0.10921	0.23283	0.28968	0.12084	-0.02653	0.33681
fusiform_R	-0.03108	-0.41872	0.20404	-0.00615	0.09555	-0.23197	-0.45067	0.07673	0.08722
hippocampus_L	-0.00108	0.14468	-0.11933	0.05802	0.15509	0.095	0.22401	-0.00996	0.29873
hippocampus_R	-0.03167	0.26532	-0.1174	-0.01516	0.09678	0.10198	0.31508	-0.01492	0.16665
inferiorparietal_L	-0.26824	0.27045	0.39515	-0.24526	0.01782	-0.62986	0.22441	0.00791	0.15917
inferiorparietal_R	-0.117	-0.4879	0.36295	-0.09847	0.26217	-0.51402	-0.46985	0.06459	0.36202
inferiortemporal_L	-0.01049	0.19023	0.32131	-0.04051	0.30242	0.52713	0.10319	0.02845	0.33105
inferiortemporal_R	-0.02301	-0.36168	0.25436	0.05603	0.30158	0.20798	-0.34574	-0.05467	0.31302
insula_L	0.04559	0.26309	-0.27348	0.03408	0.04083	0.39646	0.28951	-0.02867	0.22971
insula_R	0.23742	-0.39701	-0.0641	0.19041	0.13068	0.33766	-0.30437	0.07806	0.27953
isthmuscingulate_L	-0.09097	0.16504	-0.05782	-0.00056	0.0374	-0.69753	0.16083	-0.00811	0.17297
isthmuscingulate_R	0.04522	0.35966	-0.20874	0.0002	0.1076	-0.4864	0.25406	0.16206	-0.00394

lateraloccipital_L	-0.12778	0.30889	0.17172	-0.15308	-0.1008	-0.8297	0.14733	0.13626	-0.06281
lateraloccipital_R	-0.10903	-0.33057	0.10762	-0.08267	0.05119	-0.21429	-0.3374	0.12086	0.12467
lateralorbitofrontal_L	-0.0202	0.03473	-0.01588	0.01467	0.12855	0.25673	-0.04914	0.06676	0.09286
lateralorbitofrontal_R	0.06904	-0.53823	0.20229	0.10904	0.21088	0.03915	-0.56498	0.02998	0.15302
lingual_L	-0.15935	0.16846	0.00811	-0.17418	-0.23989	-0.9327	0.06825	0.13856	-0.16691
lingual_R	-0.0483	-0.15723	0.00024	-0.03745	0.07242	-0.84833	-0.3292	0.1465	0.07341
medialorbitofrontal_L	0.02215	-0.23481	-0.06414	0.05017	0.20002	0.46254	-0.26503	0.03888	0.10995
medialorbitofrontal_R	0.01949	-0.44054	-0.04394	0.05632	0.20245	0.49632	-0.41415	-0.00696	0.1377
middletemporal_L	-0.08578	0.13946	0.39093	-0.0904	0.21083	0.07984	0.02935	-0.04747	0.26309
middletemporal_R	0.04525	-0.45812	0.10658	0.08632	0.22402	0.03058	-0.43716	-0.02071	0.29581
normalized_gene	-0.04241	-0.07992	-0.21142	-0.02316	-0.42306	-0.59347	-0.18117	0.00041	-0.43689
pallidum_L	0.12206	0.42951	-0.36159	0.13557	-0.39249	0.56359	0.48951	-0.17817	-0.41679
pallidum_R	0.20201	0.52768	-0.30412	0.17461	-0.42434	0.67222	0.52854	-0.0955	-0.49498
paracentral_L	-0.19555	0.22885	0.19628	-0.24962	-0.0389	-0.76067	-0.09565	-0.09608	0.03279
paracentral_R	-0.17375	-0.31035	-0.02234	-0.23314	0.10458	-0.57674	-0.35111	0.00085	0.26717
parahippocampal_L	-0.03236	-0.16117	-0.07006	0.03253	-0.0317	-0.20376	-0.19506	0.01342	0.12878
parahippocampal_R	-0.06293	-0.05367	-0.03914	0.00859	0.18511	0.16039	0.02819	-0.02717	0.23347
parsopercularis_L	-0.10448	0.29924	0.40397	-0.12215	0.14255	-0.69223	0.04222	-0.00393	0.07919
parsopercularis_R	-0.10648	-0.2253	0.38323	-0.11698	0.23479	-0.55479	-0.36036	0.04575	0.24332
parsorbitalis_L	-0.16651	-0.02459	0.30333	-0.15749	0.21	-0.45992	-0.13037	-0.00811	0.11086
parsorbitalis_R	-0.02989	-0.27915	0.47718	0.04079	0.15348	-0.05899	-0.3213	-0.00951	0.26663
parstriangularis_L	-0.25081	-0.227	0.35049	-0.23646	0.0732	-0.63067	-0.37407	-0.02362	0.0571
parstriangularis_R	0.05395	-0.30919	0.1846	0.0855	0.07105	-0.23347	-0.34921	0.04585	-0.03668
pericalcarine_L	-0.09609	0.09366	0.00685	-0.12877	-0.09979	-0.61461	0.00154	0.10505	-0.11365
pericalcarine_R	-0.11611	-0.14849	0.14218	-0.10446	0.02275	-0.66694	-0.07946	0.046	0.21665
postcentral_L	-0.15114	0.44178	0.3044	-0.2162	-0.14296	-0.52841	0.16495	-0.03077	-0.10835
postcentral_R	-0.06103	-0.41304	0.08255	-0.13514	-0.13414	-0.76841	-0.58674	0.07636	-0.10346
posteriorcingulate_L	-0.1552	-0.28147	0.0949	-0.17357	0.13736	-0.38052	-0.22693	-0.031	0.16274
posteriorcingulate_R	0.03145	-0.50849	0.00587	-0.04337	0.30622	-0.55934	-0.36177	0.08803	0.31169
precentral_L	-0.10289	0.54904	0.19802	-0.15974	-0.02596	-0.60114	0.37095	-0.1246	-0.01023
precentral_R	-0.05103	-0.10215	-0.06486	-0.12259	-0.05354	-0.77078	-0.22937	-0.0043	-0.09447
precuneus_L	-0.18343	0.3295	0.21288	-0.21642	0.01525	-0.86533	0.05703	0.08706	0.04756
precuneus_R	-0.23297	-0.51878	0.29681	-0.2225	0.16807	-0.85714	-0.51024	0.13058	0.22993
putamen_L	0.14468	0.38811	-0.24111	0.13986	-0.25708	0.54705	0.52353	0.04013	-0.35958
putamen_R	0.22333	0.4671	-0.1961	0.14277	-0.207	0.61045	0.54632	-0.01375	-0.38133
rostralanteriorcingulate_L	0.12046	-0.2454	-0.31925	0.09867	0.22468	0.71173	-0.363	0.06775	0.09107
rostralanteriorcingulate_R	0.08432	-0.54124	-0.12611	0.02093	0.2509	0.39379	-0.49563	0.13517	0.22776

rostralmiddlefrontal_L	-0.06598	-0.03402	0.23187	-0.07691	0.22264	-0.06961	-0.08255	0.0987	0.06191
rostralmiddlefrontal_R	-0.09293	-0.54459	0.3235	-0.04606	0.28082	-0.06551	-0.57119	0.0801	0.26059
superiorfrontal_L	-0.04112	0.21288	0.23233	-0.07477	0.31954	0.33658	0.21693	-0.0657	0.34522
superiorfrontal_R	-0.01466	-0.65793	0.15381	0.00441	0.26561	-0.19385	-0.60717	-0.02258	0.24875
superiorparietal_L	-0.29593	0.40739	0.35278	-0.2764	0.02581	-0.45026	0.28731	0.08442	0.07962
superiorparietal_R	-0.2186	-0.59462	0.35438	-0.21694	0.14677	-0.84486	-0.59298	0.06908	0.24095
superiortemporal_L	-0.02841	0.24359	0.17818	-0.03377	0.23542	0.60047	-0.00944	-0.05809	0.32619
superiortemporal_R	0.01759	-0.66349	0.10359	0.04125	0.03911	-0.76306	-0.66901	0.03545	0.1343
supramarginal_L	-0.12093	0.39749	0.40035	-0.14805	0.205	-0.09123	0.05485	-0.00843	0.23881
supramarginal_R	-0.09223	-0.49515	0.35629	-0.12111	0.25018	-0.43586	-0.54628	0.06374	0.25243
temporalpole_L	0.04583	0.25538	-0.09489	0.05384	0.04383	0.71889	0.27135	-0.08174	0.01924
temporalpole_R	-0.1293	-0.18859	0.4036	-0.06568	0.23411	-0.58304	-0.11232	0.01164	0.27839
thalamusproper_L	0.13057	0.4497	-0.15608	0.17654	-0.28095	0.70199	0.48164	-0.12606	-0.29372
thalamusproper_R	0.06627	0.38794	-0.19983	0.101	-0.26239	0.80254	0.45807	-0.14389	-0.2677
transversetemporal_L	0.06954	0.24663	-0.391	-0.00873	-0.29057	-0.86264	0.0802	-0.03257	-0.13286
transversetemporal_R	-0.07311	-0.41036	-0.35228	-5.00E-05	-0.00583	-0.50234	-0.36224	0.07398	0.04076

ssGSEA: single sample gene set enrichment analysis; TED: thyroid eye disease

Table S4. Significant correlation between whole-brain ALFF and T cell subsets in *TED brain-blood dataset* (voxel $p < 0.005$, cluster $p < 0.05$, cluster-level GRF corrected)

Condition	Brain regions of peak voxel (DK atlas)	R value	Cluster size	MNI coordinates of peak voxel		
				X	Y	Z
B cell	superiorfrontal_L_cortex	-0.538314	17	-6	-3	63
	precentral_L_cortex	-0.563764	14	-18	-24	70
	precentral_R_cortex	-0.5956	12	10	-29	70
	paracentral_L_cortex	-0.542876	9	-5	-33	66
	lateralorbitofrontal_L_cortex	0.544508	8	-15	-27	-21
	lateralorbitofrontal_L_cortex	0.647952	7	-13	42	-27
	middletemporal_R_cortex	0.612052	7	66	-15	-15
	superiorfrontal_R_cortex	0.534211	6	9	-6	60
Monocyte	lateralorbitofrontal_R_cortex	-0.610109	32	15	45	-21
	inferiortemporal_L_cortex	-0.540265	9	-60	-39	-24
	superiorfrontal_R_cortex	0.513055	8	1	19	57
	lateraloccipital_R_cortex	0.555679	7	18	-99	21
	lateralorbitofrontal_L_cortex	-0.600476	6	-14	42	-27
	superiorfrontal_R_cortex	0.538631	6	21	16	51
DC	supramarginal_L_cortex	-0.630227	31	-36	-27	21
	insula_R_cortex	-0.618351	21	39	-18	23
	accumbensarea_R_subcortex_brainstem	-0.595797	15	9	12	-12
	rostralmiddlefrontal_L_cortex	-0.488551	10	-24	42	18
	fusiform_R_cortex	-0.594087	9	39	-33	-19
	hippocampus_R_subcortex_brainstem	-0.562232	8	24	-27	-12
	insula_R_cortex	-0.570554	8	31	-23	9
	fusiform_L_cortex	-0.471874	7	-42	-33	-18
	pallidum_L_subcortex_brainstem	-0.507252	7	-12	-3	-9
	putamen_R_subcortex_brainstem	-0.596329	7	15	6	-9
	caudate_L_subcortex_brainstem	-0.508517	7	-21	-23	23
	superiortemporal_L_cortex	-0.566451	6	-48	-6	-18
	thalamusproper_R_subcortex_brainstem	-0.562535	6	22	-21	9
	postcentral_L_cortex	0.518792	6	-45	-24	53
	postcentral_L_cortex	0.493209	6	-26	-30	63
NKT	superiorfrontal_R_cortex	-0.650652	18	24	-9	69
	pericalcarine_R_cortex	-0.548332	15	13	-99	0

	precentral_R_cortex	-0.523669	14	24	-26	72
	paracentral_R_cortex	-0.566042	7	3	-45	72
	lingual_R_cortex	-0.501785	6	1	-94	-6
	lateraloccipital_R_cortex	-0.538812	6	24	-93	6
γδT	inferiorparietal_L_cortex	0.622013	20	-42	-61	21
	superiorfrontal_R_cortex	0.593882	14	18	27	60
	postcentral_R_cortex	0.632808	13	37	-12	21
	brainstem_B_subcortex_brainstem	0.693615	7	3	-42	-42
	brainstem_B_subcortex_brainstem	0.503748	7	-6	-33	-12
	brainstem_B_subcortex_brainstem	0.500716	7	12	-27	-6
	postcentral_R_cortex	-0.512697	7	57	-22	51
	precentral_L_cortex	-0.481519	6	-45	-15	39
Central memory T cell	lateralorbitofrontal_L_cortex	0.57181	14	-12	42	-21
	lateralorbitofrontal_R_cortex	0.579819	13	15	39	-24
	caudate_R_subcortex_brainstem	0.610581	8	18	-15	27
	precentral_R_cortex	0.633964	8	54	-9	48
	accumbensarea_R_subcortex_brainstem	0.62192	7	6	6	-9
	caudalmiddlefrontal_R_cortex	0.522826	7	42	9	42
	lingual_R_cortex	0.51764	6	16	-72	-12
	accumbensarea_L_subcortex_brainstem	0.565507	6	-9	12	-9
	superiorparietal_R_cortex	0.487706	6	30	-69	54
Effector memory T cell	superiorfrontal_R_cortex	0.520925	11	15	69	0
	lateralorbitofrontal_R_cortex	0.571051	9	12	51	-27
	medialorbitofrontal_L_cortex	0.597498	7	-6	45	-20

ALFF: amplitude of low-frequency fluctuation; TED: thyroid eye disease; GRF: Gaussian random field; DK: Desikan–Killiany; MNI: Montreal Neurological Institute; DC: dendritic cell; NKT: natural killer T cell; γδT: gamma-delta T cell.

Table S5. Brain regions selected based on whole-brain ALFF and T cell subsets correlation analysis in the *TED brain-blood dataset*

ID	DK-label
8	inferiortemporal_L_cortex
11	lateralorbitofrontal_L_cortex
13	medialorbitofrontal_L_cortex
16	paracentral_L_cortex
23	precentral_L_cortex
27	superiorfrontal_L_cortex
30	supramarginal_L_cortex
51	lateraloccipital_R_cortex
52	lateralorbitofrontal_R_cortex
54	medialorbitofrontal_R_cortex
62	postcentral_R_cortex
64	precentral_R_cortex
68	superiorfrontal_R_cortex
77	caudate_R_subcortex_brainstem
78	putamen_R_subcortex_brainstem
79	pallidum_R_subcortex_brainstem
80	accumbensarea_R_subcortex_brainstem
83	brainstem_B_subcortex_brainstem

ALFF: amplitude of low-frequency fluctuation; TED: thyroid eye disease; DK: Desikan–Killiany.

References

[1] H. Zhang, Y. Liu, D. Xia, M. Jiang, Y. Li, J. Sun, H. Guan, L. Zhu, X. Song, J. Wang, X. Fan, H. Zhou, "The insular cortex is not insular in thyroid eye disease: Neuroimaging revelations of central–peripheral system interaction," *Journal of Neuroinflammation* **21** (2024): 51. https://doi.org/10.1186/s12974-024-03044-4

[2] T. T. Liu, M. Falahpour,"Vigilance effects in resting-state fMRI," *Frontiers in Neuroscience* **14** (2020): 321. https://doi.org/10.3389/fnins.2020.00321

[3] C.-G. Yan, X.-D. Wang, X.-N. Zuo, Y.-F. Zang, "DPABI: Data Processing & Analysis for (Resting-State) Brain Imaging," *Neuroinformatics* **14** (2016): 339–351. https://doi.org/10.1007/s12021-016-9299-4

[4] Y.-F. Zang, Y. He, C.-Z. Zhu, Q.-J. Cao, M.-Q. Sui, M. Liang, L.-X. Tian, T.-Z. Jiang, Y.-F. Wang, "Altered baseline brain activity in children with ADHD revealed by resting-state functional MRI," *Brain and Development* **29** (2007): 83–91. https://doi.org/10.1016/j.braindev.2006.07.002

[5] M. J. Hawrylycz, E. S. Lein, A. L. Guillozet-Bongaarts, E. H. Shen, L. Ng, J. A. Miller, et al., "An anatomically comprehensive atlas of the adult human brain transcriptome," *Nature* **489** (2012): 391–399. https://doi.org/10.1038/nature11405

[6] A. Arnatkeviciute, B. D. Fulcher, A. Fornito, "A practical guide to linking brain-wide gene expression and neuroimaging data," *NeuroImage* **189** (2019): 353–367. https://doi.org/10.1016/j.neuroimage.2019.01.011

[7] J. Quackenbush, "Microarray data normalization and transformation," *Nature Genetics* **32** (2002): 496–501. https://doi.org/10.1038/ng1032

[8] M. Hawrylycz, J. A. Miller, V. Menon, D. Feng, T. Dolbeare, et al., "Canonical genetic signatures of the adult human brain," *Nature Neuroscience* **18** (2015): 1832–1844. https://doi.org/10.1038/nn.4171

[9] B. D. Fulcher, M. A. Little, N. S. Jones, "Highly comparative time-series analysis: The empirical structure of time series and their methods," *Journal of the Royal Society Interface* **10** (2013): 20130048. https://doi.org/10.1098/rsif.2013.0048
